# CD4^+^ T cells contribute to neurodegeneration in Lewy body dementia

**DOI:** 10.1126/science.abf7266

**Published:** 2021-10-14

**Authors:** David Gate, Emma Tapp, Olivia Leventhal, Marian Shahid, Tim J. Nonninger, Andrew C. Yang, Katharina Strempfl, Michael S. Unger, Tobias Fehlmann, Hamilton Oh, Divya Channappa, Victor W. Henderson, Andreas Keller, Ludwig Aigner, Douglas R. Galasko, Mark M. Davis, Kathleen L. Poston, Tony Wyss-Coray

**Affiliations:** 1 Department of Neurology, Northwestern University, Chicago, Illinois, USA.; 2 Department of Neurology and Neurological Sciences, Stanford University School of Medicine, Stanford, California, USA.; 3 Wu Tsai Neurosciences Institute, Stanford University, Stanford, CA, USA.; 4 Department of Bioengineering, Stanford University, Stanford, California, USA.; 5 Chemistry, Engineering, and Medicine for Human Health (ChEM-H), Stanford University, Stanford, California, USA.; 6 Institute of Molecular Regenerative Medicine, Paracelsus Medical University, Salzburg, Austria.; 7 Spinal Cord Injury and Tissue Regeneration Center Salzburg, Paracelsus Medical University, Salzburg, Austria; 8 QPS Austria GmbH, Parkring 12, 8074 Grambach, Austria; 9 Chair for Clinical Bioinformatics, Saarland University, Saarbrucken, Germany.; 10 Department of Neurosciences, University of California, San Diego, La Jolla, California, USA.; 11 Department of Microbiology and Immunology, School of Medicine, Stanford University, Stanford, California, USA.; 12 Howard Hughes Medical Institute, Stanford University School of Medicine, Stanford, California, USA.

## Abstract

Recent studies indicate that the adaptive immune system plays a role in Lewy body dementia (LBD). However, the mechanism regulating T cell brain homing in LBD is unknown. Here, we observed T cells adjacent to Lewy bodies and dopaminergic neurons in post-mortem LBD brains. Single-cell RNA sequencing of cerebrospinal fluid (CSF) identified upregulated expression of *C-X-C Motif Chemokine Receptor 4* (*CXCR4*) in CD4^+^ T cells in LBD. CSF protein levels of the CXCR4 ligand, C-X-C Motif Chemokine Ligand 12 (CXCL12) were associated with neuroaxonal damage in LBD. Furthermore, we observed clonal expansion and upregulated *Interleukin 17A* expression by CD4^+^ T cells stimulated with a phosphorylated α-synuclein epitope. Thus, CXCR4-CXCL12 signaling may represent a mechanistic target for inhibiting pathological interleukin-17-producing T cell trafficking in LBD.

## Summary

The immune system is implicated in the neurodegenerative process of Lewy body dementia.

Lewy body dementia (LBD) encompasses two disorders characterized by abnormal deposits of α-synuclein in the brain: dementia with Lewy bodies (DLB) and Parkinson’s disease dementia (PDD). PDD is defined by changes in memory and behavior and afflicts patients in late stage Parkinson’s disease (PD) (1). The symptoms and cognitive profiles of DLB and PDD are highly similar (2). Several lines of evidence suggest involvement of the adaptive immune system in DLB (3) and PDD (4–6). Immune alterations have been reported in the peripheral blood of PD patients, including changes to lymphocyte activation (7–9). The involvement of CD4^+^ T cells in PD is supported by studies in mouse models (10–13) and in vitro culture systems (6). Moreover, recent studies have found that a defined set of peptides derived from α-synuclein act as antigenic epitopes and promote T cell responses in non-demented PD patients ex vivo (4, 5, 14). However, showing a role for T cells in the neurodegenerative process of LBD in vivo is lacking. Furthermore, the mechanism regulating T cell brain homing in LBD remains unknown.

## Results

### Neurodegeneration in LBD study subjects

To assess adaptive immunity in LBD, we integrated analyses of multiple cohorts consisting of healthy aged controls (n=162) and patients with clinical DLB and PD (collectively referred to as PD-DLB; n=148) (Fig. S1A, Table S1 and Data S1). Montreal Cognitive Assessment scores indicated reduced cognition in PD-DLB subjects (*P* = 8.6X10^−5^; Fig. S1B). Furthermore, proteomic analysis of cerebrospinal fluid (CSF) indicated increased levels of neurofilament light chain (NEFL; *P* = 0.0031; Fig. S1C). NEFL reflects neuronal damage in a variety of neurological disorders (12–14). Because PD has a long prodromal phase before dementia onset, we stratified patients as PD-not cognitively impaired (PD-NCI) or PDD (those with cognitive impairments and dementia). Compared to healthy subjects, patients diagnosed as PDD (*P* = 6.33X10^−13^) and DLB (*P* = 4.02X10^−13^) presented with lower cognitive scores than PD-NCI patients (*P* = 0.83; Fig. S1D). These data suggest increased neurodegeneration in our PDD and DLB subjects.

### T cells home to the LBD brain and reside in close proximity to α-synuclein deposits

**Fig. 1.**
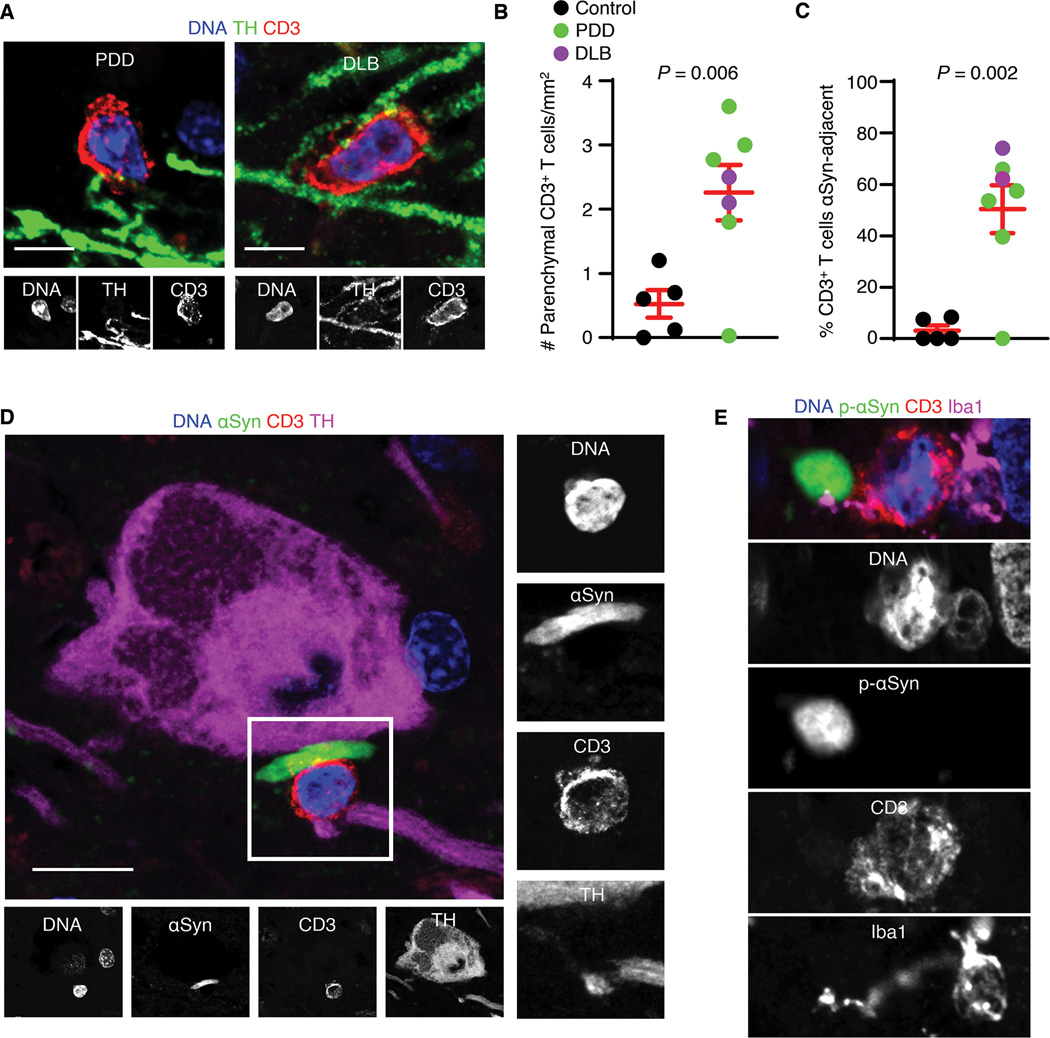
T cells localize to dopaminergic neurons and α-synuclein deposits in the LBD brain. (A) Confocal images of parenchymal CD3^+^ T cells adjacent to TH^+^ neuronal processes in PDD and DLB substantia nigra. Scale bars = 10 μm. CD3^+^ T cells were detected in 6/7 LBD brains analyzed. (B) Quantification of parenchymal CD3^+^ T cells reveals higher numbers of T cells in LBD vs. healthy substantia nigra. Data are mean ± SEM. (C) Quantification of percent parenchymal CD3^+^ T cells adjacent to α-synuclein deposits in LBD brains. Cells determined to be adjacent to α-synuclein deposits were within 5μm distance. Data are mean ± SEM. (D) Confocal image of PDD substantia nigra showing a CD3^+^ T cell in close proximity to an α-synuclein^+^ Lewy neurite. Scale bar = 10 μm. Similar results were observed in 6/7 LBD brains. (E) An Iba1^+^ innate immune cell in the PDD substantia nigra. Note the Iba1^+^ process appearing to contact the CD3^+^ T cell and α-synuclein^+^ Lewy body in PDD. Scale bar = 5 μm. Similar results were observed in 6/7 LBD brains.

We next examined post-mortem substantia nigra to localize and quantify T cells in LBD. Immunohistochemical analysis showed CD3^+^ T cells in close proximity to neuronal processes labeled by the dopamine enzyme tyrosine hydroxylase (TH) in the substantia nigra of PDD and DLB brains (Fig. 1A and S2A). Quantification of control (non-neurologic disease) and LBD substantia nigra indicated higher numbers of CD3^+^ T cells in LBD (Welch’s t-test, *P* = 0.006; Fig. 1B). We then probed LBD brains for α-synuclein to determine whether T cells localize to these protein deposits. Indeed, we found CD3^+^ T cells adjacent to α-synuclein deposits in LBD brains (Fig. S2B and C). Quantification of these cells revealed a higher percentage of CD3^+^ T cells localized to α-synuclein deposits in LBD substantia nigra (Welch’s t-test, *P =* 0.002; Fig. 1C). We also detected CD3^+^ T cells adjacent to Lewy neurites surrounding TH^+^ neurons in PDD (Fig. 1D and Fig. S3A and B) and DLB substantia nigra (Fig. S3C). CD3^+^ T cells were also found near α-synuclein^+^ Lewy bodies adjacent to vesicular glutamate transporter 1 (vGLUT1)^+^ glutamatergic neurons in the hippocampal CA2 region (Fig. S3D). Notably, CD3^+^ T cells were also bound to Iba1^+^ innate immune cells, which extended processes towards phosphorylated α-synuclein^+^ Lewy bodies in PDD (Fig. 1E) and DLB (Fig. S3E and F). In mice expressing human α-synuclein (Thy1-αSyn), CD3^+^ T cells were found adjacent to α-synuclein deposits in the midbrain (Fig. S4). Thus, T cells home to the LBD brain and reside in close proximity to α-synuclein deposits.

### *CXCR4* is upregulated in CD4^+^ T cells in LBD CSF

**Fig. 2.**
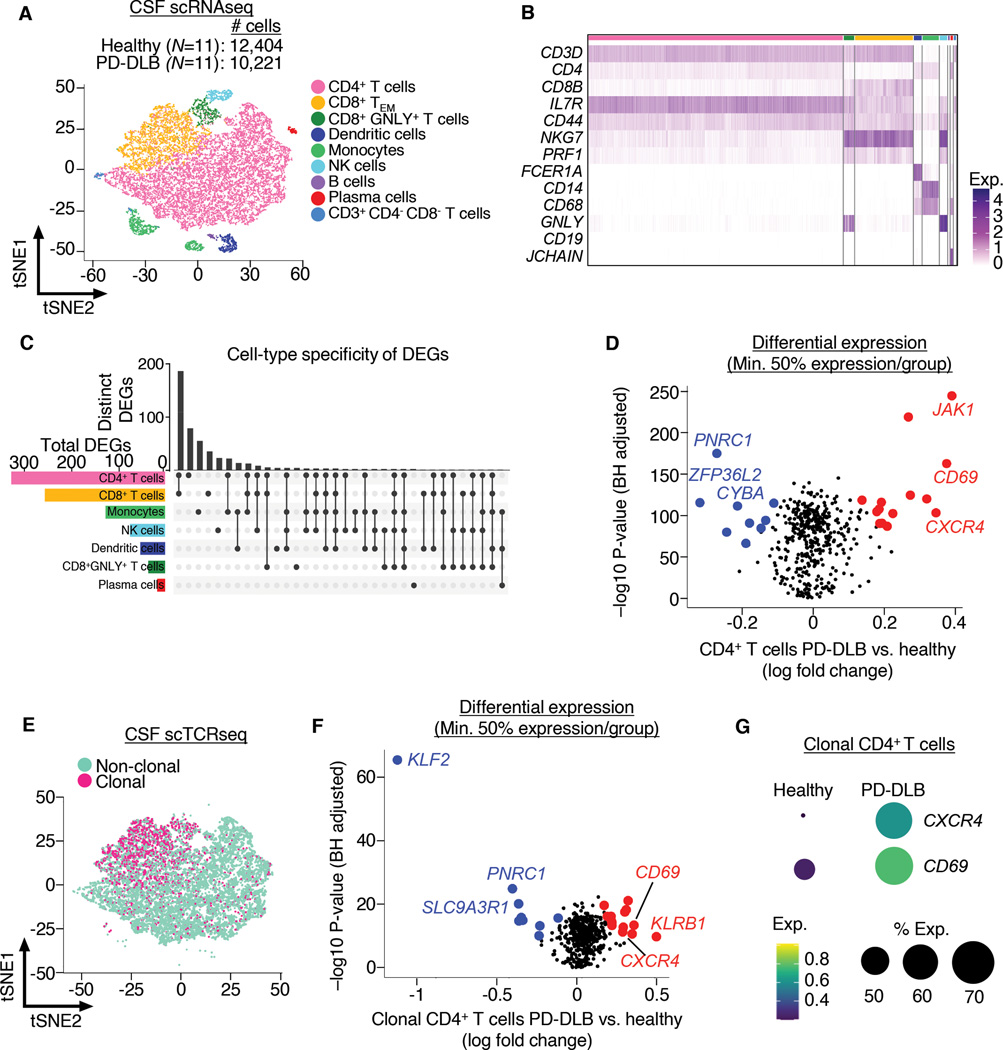
Upregulated *CXCR4* demarks CD4^+^ T cells in PD-DLB. (A) scRNAseq of CSF cells shows clusters of various types of immune cells by tSNE. (B) Marker expression of CSF immune cells used to classify clusters. (C) UpSet plot showing cell-type specific analysis of differentially expressed genes of PD-DLB vs. healthy CSF immune cells indicating the highest number of differentially expressed genes in CD4^+^ T cells. (D) Volcano plot showing differentially expressed genes of CD4^+^ T cells from LBD vs. healthy CSF. Note the increased expression of *CXCR4* in LBD. (E) scTCRseq of CSF immune cells showing clonal vs. non-clonal T cells plotted by tSNE. (F) Volcano plot of differential expression analysis of clonal CD4^+^ T cells showing increased expression of *CD69*, *KLRB1* and *CXCR4* in PD-DLB vs. healthy CSF. (G) Dot plot showing higher levels of *CXCR4* and *CD69* in PD-DLB vs. healthy CSF clonal CD4^+^ T cells.

To uncover potential mechanisms of brain entry in LBD, we performed single-cell RNA sequencing (scRNAseq) (15, 16) of CSF cells isolated from age- and sex-matched healthy (n=11) and PD-DLB (n=11) subjects (Fig. S5A). Multidimensional reduction of scRNAseq data by t-distributed stochastic neighbor embedding (tSNE) revealed clusters of immune cells (Fig. 2A). Clusters expressed marker genes corresponding to each immune cell subtype (Fig. 2B) and were not specific to group or sex (Fig. S5B). Cell-type specific differential expression of PD-DLB vs. healthy CSF cell clusters revealed CD4^+^ T cells as the most transcriptionally altered immune cell subtype (Fig. 2C and Data S2). Highly differentially expressed PD-DLB CD4^+^ T cell genes included *Janus kinase 1* (*JAK1*), a kinase essential for cytokine signaling, and the T cell activation gene *Cluster Of Differentiation 69* (*CD69*) (Fig. 2D). The chemokine receptor gene *C-X-C Motif Chemokine Receptor 4* (*CXCR4*) was also highly upregulated in PD-DLB CD4^+^ T cells (Fig. 2D). Moreover, *CXCR4* and *CD69* were highly expressed by the majority of CD4^+^ T cells (Fig. S5C). Quantification of individual subjects’ CD4^+^ T cell *CXCR4* and *CD69* expression revealed higher levels in PD-DLB vs. healthy CSF (Welch’s t-test, *P* = 0.03 and *P* = 0.025, respectively; Fig. S5D). Analysis of pathways containing *CXCR4* indicated altered metabolic and catalytic activity and response to cytokine stimulus in CD4^+^ T cells in PD-DLB (Fig. S5E). Thus, enhanced CD4^+^ T cell cytokine signaling and activation can be observed in PD-DLB CSF.

The increase in activation of CSF CD4^+^ T cells in PD-DLB prompted us to determine whether clonally expanded (i.e. antigen-specific) cells were distinct in PD-DLB. To assess clonal expansion, we performed single-cell T cell receptor sequencing (scTCRseq) on the same CSF cells as above (Fig. 2E). Comparing RNA transcriptomes of clonal CD4^+^ T cells from healthy and PD-DLB CSF by differential expression again showed increased expression of *CD69* and *CXCR4* in PD-DLB (Fig. 2F and G and Data S3). Clonal T cells were not specific to disease group or sex (Fig. S6A). Pathway analysis of differentially expressed clonal CD4^+^ PD-DLB T cell genes revealed regulation of cytokine-mediated signaling and intracellular signal transduction as the most altered pathways containing *CXCR4* (Fig. S6B). We also detected higher expression of *Killer cell lectin-like receptor subfamily B, member 1* (*KLRB1*), a marker of pro-inflammatory IL-17-producing (Th17) memory CD4^+^ T cells (17, 18) (Fig. 2F and Data S3). We also localized CD3^+^KLRB1^+^ T cells to phosphorylated α-synuclein deposits in the parenchyma of PDD brains (Fig. S6C). Thus, LBD may involve enhanced activation of pro-inflammatory CD4^+^ Th17 cells.

### The CXCR4 ligand CXCL12 is associated with neurodegeneration in LBD

**Fig. 3.**
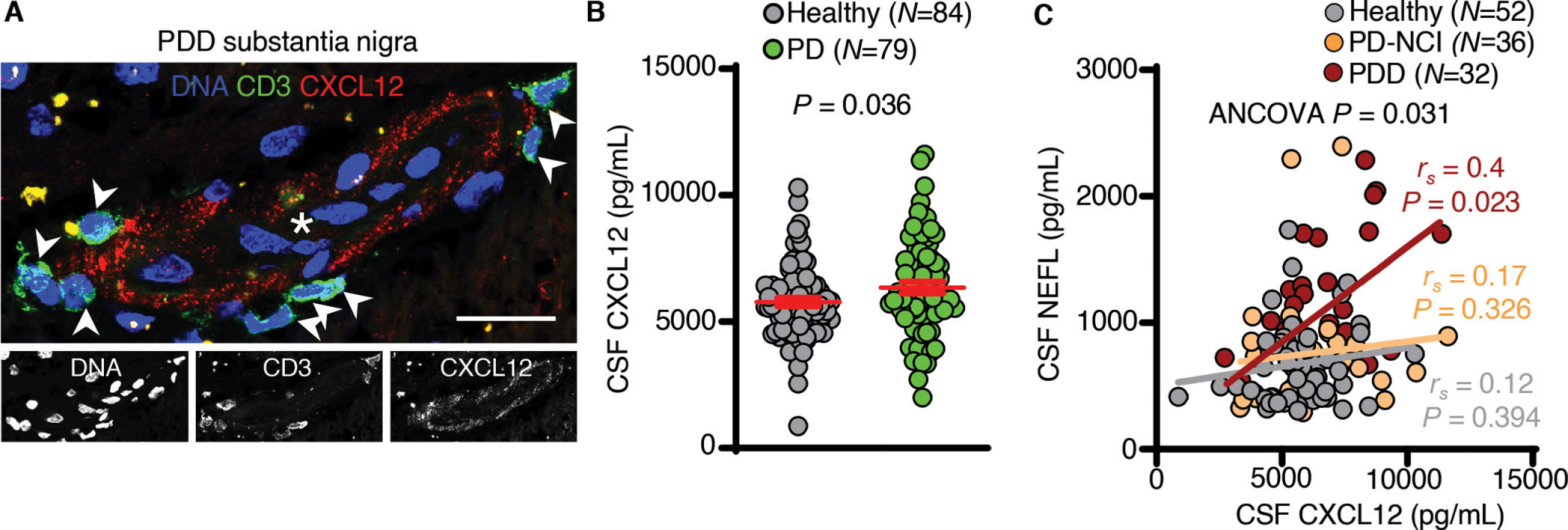
CXCL12 is associated with neurodegeneration in LBD. (A) A PDD substantia nigra brain blood vessel showing localization of CXCL12 to the cerebral vasculature. Arrowheads indicate CD3^+^ T cells in the perivascular space. Asterisk indicates blood vessel lumen. Scale bar = 50 μm. Similar results were observed in 7/7 LBD brains. (B) Single molecule array measurement of CXCL12 indicating higher levels in PD vs. healthy CSF. Data are mean ± SEM. (C) Regression analysis correlating CSF CXCL12 and NEFL levels in healthy, PD-NCI and PDD. Note the significant correlation of CXCL12 and NEFL in PDD but not PD-NCI or healthy CSF.

To determine if T cells express CXCR4 in the brain, we performed immunohistochemistry on PDD meninges, which revealed meningeal CD3^+^CXCR4^+^ cells (Fig. S7A). We noted localization of the CXCR4 ligand, C-X-C Motif Chemokine Ligand 12 (CXCL12) to CD3^+^CXCR4^+^ cells in the meninges (Fig. S7A). In mice, CXCL12 is expressed by cerebrovascular endothelial cells and promotes recruitment of CD4^+^ T cells (19). Within the PDD brain, CXCL12 localized to the cerebrovasculature (Fig. 3A), confirmed by co-staining PDD brains with the vascular marker Cluster Of Differentiation 31 (CD31; Fig. S7B). CD3^+^ T cells resided in the perivascular space adjacent to CXCL12^+^ vessels (Fig. 3A and Fig. S7C).

We next sought to determine whether levels of CSF CXCL12 were associated with cognitive impairment in PD. We measured CXCL12 in a cohort of age- and sex-matched healthy (n=84) and PD (n=79) subjects (Fig. S8A). This revealed higher levels of CSF CXCL12 in PD (Welch’s t-test, *P* = 0.036; Fig. 3B). We separated this PD cohort by clinical diagnoses as PD-NCI or PDD, which revealed lower cognitive scores in PDD subjects compared to healthy (*P* = 3.12X10^−11^) and PD-NCI (*P* = 7.67X10^−10^) subjects (one-way ANOVA (F (2,135) = 31.697, *P* = 5.18X10^−12^); Fig. S8B). NEFL levels also distinguished PDD from healthy (*P* = 1.00X10^−4^) and PD-NCI (*P* = 8.30X10^−3^) subjects (one-way ANOVA, (F (2,117) = 9.161, *P* = 0.0002); Fig. S8C). Age did not significantly impact CXCL12 levels in this cohort (ANCOVA, (F (2,150) = 2.867, *P* = 0.071); Fig. S8D). We then correlated CXCL12 levels with neurodegenerative disease biomarkers, including ubiquitin carboxyl-terminal esterase L1, total tau, phosphorylated tau 181, amyloid-β, α-synuclein and NEFL (Fig. S8E). CXCL12 levels correlated most positively with NEFL in PDD (r_*s*_ = 0.40; *P* = 0.023), and these correlations were lesser in healthy (r_*s*_ = 0.12; *P* = 0.394) and PD-NCI (r_*s*_ = 0.17; *P* = 0.326) subjects (ANCOVA (F (2,114) = 3.484, *P* = 0.031); Fig. 3C). Thus, dysregulated CXCR4-CXCL12 signaling is associated with neurodegeneration in LBD.

### *CXCR4* demarks CD4^+^ T cells that are unique to the CSF

**Fig. 4.**
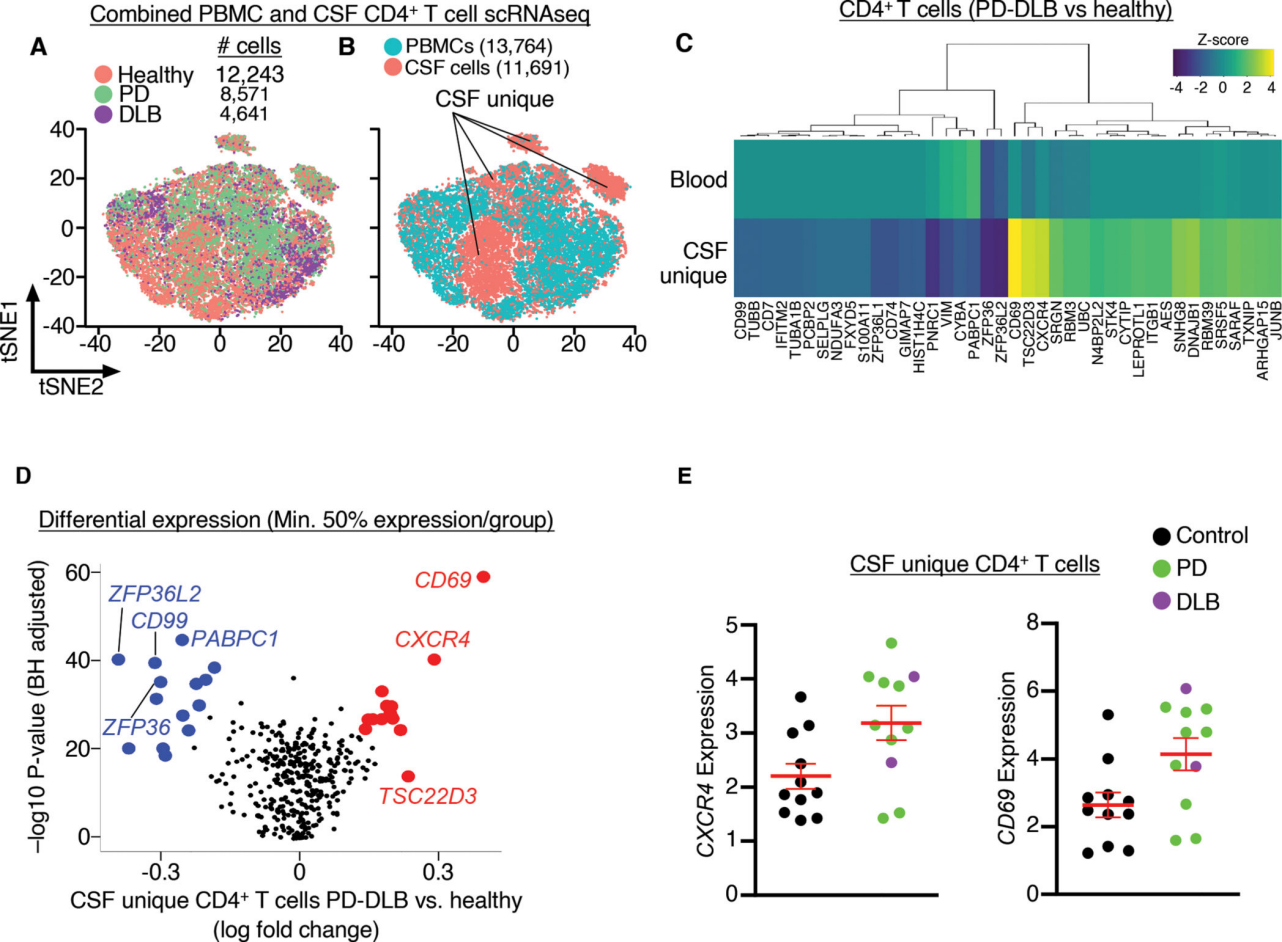
CXCR4 demarks CD4^+^ T cells that are unique to the CSF in LBD. (A) tSNE plot showing overlayed distribution of peripheral versus CSF CD4^+^ T cells from healthy, PD and DLB subjects. (B) tSNE plot showing clusters of CD4^+^ T cells that are unique to the CSF. (C) Hierarchical clustering of standardized z-scores comparing PD-DLB to healthy CD4^+^ T cells from PBMCs and CSF. Note the clustering of genes *CXCR4*, *CD69* and *TSC22D3* that demark CSF unique CD4^+^ T cells. (D) Volcano plot showing differential expression analysis comparing PD-DLB to healthy CSF unique CD4^+^ T cells. (E) Quantification of individual subjects’ *CXCR4* and *CD69* expression of PD-DLB versus healthy CSF unique CD4^+^ T cells showing higher expression of each gene in PD-DLB. Data are mean ± SEM.

Because peripheral T cells have been shown to be dysregulated in PD (4, 5, 14), we compared CD4^+^ T cells of the peripheral immune system and CSF. We performed scRNAseq on peripheral blood mononuclear cells (PBMCs) of the same subjects we analyzed by CSF scRNAseq and focused our analysis on CD4^+^ T cells (Fig. 4A). We uncovered CD4^+^ T cell populations that were unique to the CSF (referred to as CSF unique; Fig. 4B). We also identified upregulated *CXCR4*, *CD69*, and *TSC22D3* as the primary genes defining CSF unique T cells (Fig. 4D). Quantification of individual subjects’ CSF unique CD4^+^ T cell *CXCR4* and *CD69* expression revealed higher levels in PD-DLB vs. healthy CSF (Welch’s t-test, *P* = 0.0218 and *P* = 0.0217, respectively; Fig. 4E). Thus, *CXCR4* may regulate homing of CD4^+^ T cells to the LBD brain.

### α-synuclein stimulation drives T cell clonal expansion and activation

Our immunohistochemistry results indicated close proximity of T cells with α-synuclein in LBD brains. This led us to investigate whether α-synuclein could drive T cell clonal expansion and activation. Several peptides derived from α-synuclein act as antigenic epitopes and promote T cell responses in PD PBMCs (4, 5). We incubated PBMCs from healthy (n=32) and PD (n=53) subjects with a pool of eight antigenic α-synuclein peptides and measured activation of CD3^+^ T cells by flow cytometry using co-expression of HLA-DR and CD38 (Fig. S9A). Surprisingly, control PD patient T cells in the absence of stimulation exhibited higher percentages of HLA-DR^+^CD38^+^ T cells than healthy subjects (Welch’s t-test, *P* = 0.006; Fig. S9B and C). This suggests higher baseline levels of peripheral T cell activation exists in PD patients in vivo. We also detected higher levels of T cell activation following stimulation with the α-synuclein peptide pool (Welch’s t-test, *P* = 0.002; Fig. S10C). We confirmed increased activation of PD T cells following α-synuclein stimulation by measuring CD69 by flow cytometry (Welch’s t-test, *P* = 0.027; Fig. S9D).

**Fig. 5.**
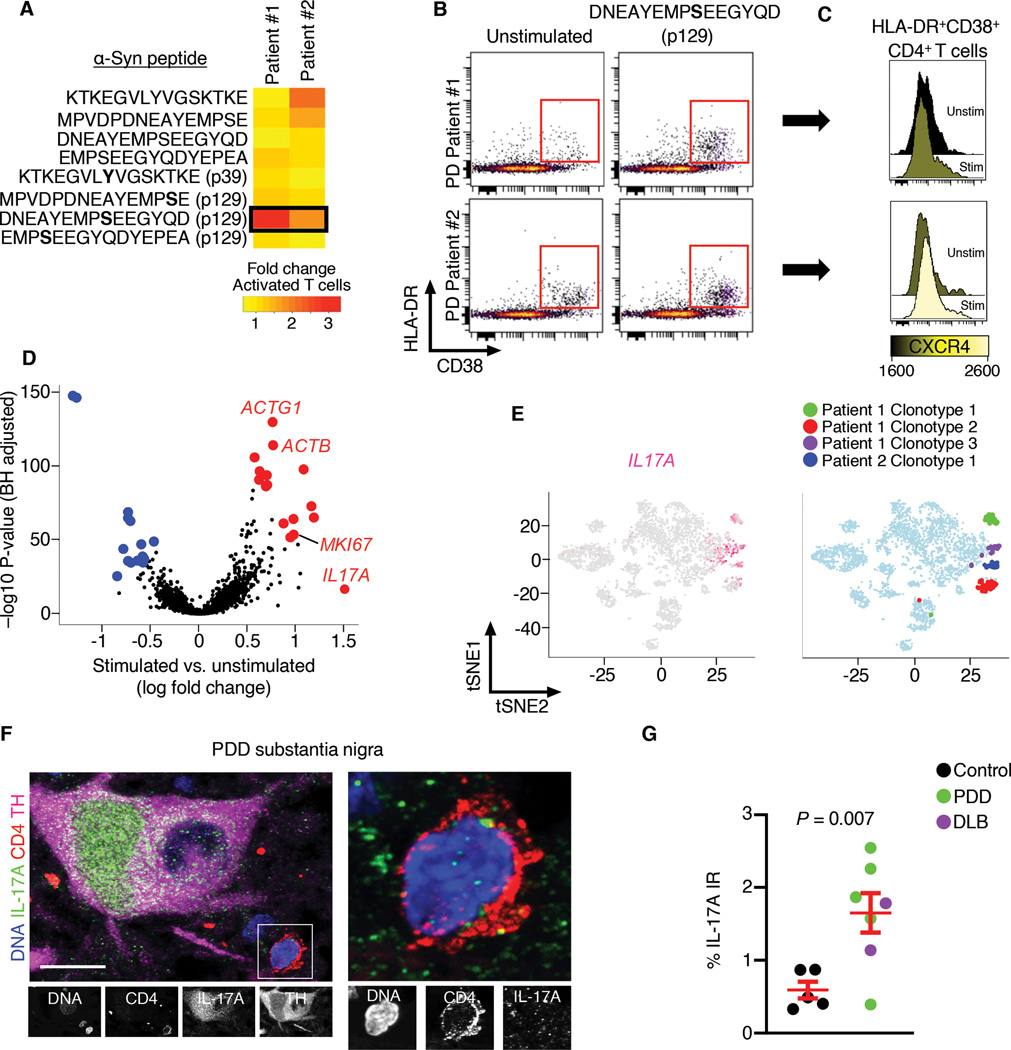
Stimulation of LBD T cells with α-synuclein promotes *IL-17A* expression. (A) Heatmap showing fold change of T cell activation (% HLA-DR^+^CD38^+^ CD3^+^ T cells) between unstimulated and stimulated PBMCs. Cells were incubated with α-synuclein peptides known to be antigenic. Note that peptide DNEAYEMPSEEGYQD (p129) increased T cell activation in patients #1 and #2. (B) Flow cytometry plots of unstimulated and DNEAYEMPSEEGYQD (p129)-stimulated cells showing increased T cell activation (% HLA-DR^+^CD38^+^ CD3^+^ T cells) by the α-synuclein peptide. (C) Histograms showing increased expression of CXCR4 in DNEAYEMPSEEGYQD (p129)-stimulated HLA-DR^+^CD38^+^CD4^+^ T cells in both patients by flow cytometry. (D) Differential expression analysis of stimulated vs. unstimulated HLA-DR^+^CD38^+^CD3^+^ T cells shows increased expression of antigen-dependent T cell activation genes *ACTG1* and *ACTB*, the proliferative gene *MKI67*, and the pro-inflammatory cytokine *IL17A*. (E) tSNE plots indicating overlap of cells expressing *IL17A* and clonally expanded T cells (clonotypes) from both patients. (F) Confocal images of control (non-neurologic disease) and PDD post-mortem brains showing CD4^+^IL-17A^+^ T cells adjacent to an IL-17A^+^TH^+^ neuron in the PDD substantia nigra. Scale bar = 10 μm. (G) Quantification of IL-17A immunoreactivity (IR) in the substantia nigra of control and LBD brains showing increased IL-17A in LBD. Similar results were observed in 6/7 LBD brains. Data are mean ± SEM.

To identify patient-specific antigenic α-synuclein peptides, we selected two PD patients who exhibited appreciable increases in T cell activation by the α-synuclein peptide pool (Fig. S9E). We then measured T cell activation in these subjects using individual α-synuclein peptides. This strategy revealed activation of T cells by the peptide DNEAYEMPSEEGYQD containing a phosphorylated serine residue at amino acid position 129 (Fig. 5A and B). HLA-DR^+^CD38^+^CD4^+^ T cells upregulated CXCR4 in response to peptide stimulation (Fig. 5C). To determine transcriptional changes induced by this peptide, we sorted activated T cells from unstimulated and stimulated PBMCs (Fig. S10A). We then performed scRNAseq on HLA-DR^+^CD38^+^ T cells to interpret transcriptomic alterations (Data S4). Stimulated T cells had increased expression of *Actin Gamma 1* (*ACTG1*) and *Actin Beta* (*ACTB*) (Fig. 5D), which regulate cytoskeletal control of antigen-dependent T cell activation (20). We also noted increased expression of *Marker Of Proliferation Ki-67*, suggesting increased proliferation of stimulated T cells (Fig. 5D).

### Th17 cell involvement in the degeneration of neurons in LBD

Notably, we also detected higher expression of *Interleukin 17A* (*IL17A)* in cells stimulated with α-synuclein (Fig. 5D). *IL17A* encodes the pro-inflammatory cytokine IL-17, which is secreted by Th17 cells (21). To determine whether *IL17A*-expressing cells were clonally expanded, we performed scTCRseq on stimulated and unstimulated cells. This revealed clonal populations from stimulated cells of both patients (Fig. S10B). We then plotted *IL17A* expression by tSNE and identified clonally expanded TCRs (clonotypes) from each subject (Fig. 5E). *IL17A*-expressing cells co-expressed *CD4* and some clonotypes also expressed the Th17-associated cytokine gene *Interleukin 22* (*IL22*; Fig. S10C). We confirmed the presence of CD4^+^IL-17A^+^ T cells in the PDD substantia nigra, which were adjacent to TH^+^IL-17A^+^ neurons (Fig. 5F). We also detected higher levels of IL-17A immunoreactivity in LBD brains (Welch’s t-test, *P* = 0.007; Fig. 5G). Public datasets revealed lack of *IL17A* RNA expression in the brain, yet the gene encoding the IL17A receptor, *IL17RA*, was highly expressed in the midbrain (Fig. S11A), suggesting an external source of IL17A protein in neurons. Public histology data indicated an age-dependent accumulation of IL17A in neurons (Fig. S11B). Finally, to confirm IL17A antibody specificity, we pre-incubated antibodies with recombinant IL-17A, which ablated IL-17A immunoreactivity (Fig. S11C).

## Discussion

In conclusion, these results implicate Th17 cell involvement in the degeneration of neurons in LBD. Notably, CXCR4 regulates cell migration (22), and antagonism of CXCR4 modulates the pathogenicity of Th17 cells (23). Thus, our investigation of intrathecal immunity uncovered the CXCR4-CXCL12 signaling axis as a potential therapeutic target for LBD. Several CXCR4 antagonists are currently approved for clinical use to treat a wide variety of diseases (24–30). Given the safety, bioavailability and tolerability of CXCR4 antagonists (26), these drugs could be utilized to inhibit trafficking of pathological Th17 cells into the LBD brain. Finally, we identified an antigenic α-synuclein epitope that promoted expression of *IL17A*, a pro-inflammatory cytokine involved in autoimmune diseases (21). In animal models of neurodegenerative disease, Th17 cells play a direct role in neuronal loss (31, 32). Moreover, human Th17 cells have been shown to promote blood-brain barrier disruption and central nervous system inflammation via IL-17A (33). Thus, our study provides a mechanism for Th17 cell-mediated dopaminergic cell death via secretion of inflammatory IL-17A, thereby implicating autoimmunity in LBD (Fig. S12).

## Supplementary Material

Supplementary Material

Data S3

Data S2

Data S1

Data S4

## Data and materials availability:

The scRNAseq datasets analyzed during the current study are available in the Gene Expression Omnibus repository under accession numbers GSE141578 and GSE161192.
