# Supplementary material for: CD4+ T cells contribute to neurodegeneration in Lewy body dementia
Source: Science. Author manuscript; Available in PMC 2022 May 20. (PMC9122025; doi:10.1126/science.abf7266)
Supplement: Supplementary Material [file NIHMS1780000-supplement-Supplementary_Material.docx]

Supplementary Materials for

CD4^+^ T cells contribute to neurodegeneration in Lewy body dementia

David Gate, Emma Tapp, Olivia Leventhal, Marian Shahid, Tim J. Nonninger, Andrew C. Yang, Katharina Strempfl, Michael S. Unger, Tobias Fehlmann, Hamilton Oh, Divya Channappa, Victor W. Henderson, Andreas Keller, Ludwig Aigner, Douglas R. Galasko, Mark M. Davis, Kathleen L. Poston, Tony Wyss-Coray

Correspondence to: dgate@northwestern.edu; twc@stanford.edu

**This PDF file includes:**

Materials and Methods

Figs. S1 to S12

Table S1

Captions for Data S1 to S4

**Other Supplementary Materials for this manuscript include the following:**

Data S1 to S4

Data S1- Study Subjects and Biomarker Data

Data S2- CSF Immune Cell Differential Expression

Data S3- CSF Clonal CD4 T Cell Differential Expression

Data S4- Alpha Synuclein Stimulation Differential Expression

Materials and Methods

Study Participants and Biomarkers

Samples were acquired through the NIA funded Stanford Alzheimer’s Disease Research Center (ADRC) and the University of California San Diego ADRC. Collection of brain tissue, PBMCs and CSF was approved by the Institutional Review Board of each university, and written consent was obtained from all participants. A total of 310 participants were used in this study. Diagnostic group was determined during clinical consensus meeting after each participant completed a formal neurological and motor examination, comprehensive neuropsychological battery and clinical history by a licensed neurologist. All participants were adjudicated as PD, DLB or healthy based on published criteria. For PD, we used the UK PD Brain Bank Criteria (*34*). For DLB, we used the 4^th^ Consensus report for DLB Diagnostic Criteria (*35*). Cognitively impaired PDD participants were determined to have mild cognitive impairment (*36*) or dementia (*37*) based on published criteria. All participants were free from acute infectious diseases and in good physical condition. Group characteristics, including demographic, clinical and biomarker data for each participant are presented in Table S1 and Data S1. Biomarkers I included CXCL12 and α-synuclein. Biomarkers II included UCHL1, tau, phosphorylated tau, NEFL, Aβ_40_ and Aβ_42_. Biomarkers were measured using Simoa technology by Quanterix.

Tissue Collection

PBMCs were isolated from blood by layering diluted blood (1:1 in PBS) on top of an equal volume of Ficoll, followed by centrifugation and isolation of the buffy coat. CSF was collected by lumbar puncture, then centrifuged at 300G to pellet immune cells. CSF samples were checked for blood contamination by resuspending the pelleted cells in 100 μl of CSF and mixing 10 μl (10%) CSF with 10 μl trypan blue to assess red blood cell content and viability. Cells were visualized on a TC20 automated cell counter (BioRad) and cell viability and presence/absence of red blood cells was recorded. CSF samples contaminated with blood were not used in the study. The resuspended cells were then mixed with 900 μl Recovery Cell Culture Freezing Medium (Thermo Fisher). All samples were frozen overnight at -80°C in a Mr. Frosty freezing container (Thermo Fisher) and transferred the following day to liquid nitrogen for storage.

Immunohistochemistry and confocal microscopy of human tissues

Paraffin-embedded brain tissues of the substantia nigra or meninges were sectioned at 5μm thickness. Deparaffinization was achieved by washing slides through a series of xylenes and decreasing concentrations of ethanol. Tissue sections were then subjected to antigen retrieval using citrate buffer, pH 6.0 (Sigma-Aldrich) at 95° C for 30 min. Following rinsing with PBS, sections were incubated in blocking buffer containing PBS with 10% normal donkey serum and 0.03% Triton-X (Sigma-Aldrich) for 2 hours at room temperature. Slides were then incubated with primary antibody in blocking buffer overnight at 4 °C. The following day, slides were rinsed with PBS then incubated in appropriate Alexa Fluor secondary antibodies (Thermo Fisher). Sections were then rinsed and stained with Hoechst DNA dye (Thermo Fisher) prior to being coverslipped with ProLong mounting medium (Invitrogen). Primary antibodies included rat anti-human CD3 (Abcam; ab11081; RRID:AB_2889189), rabbit anti-CD3 (Cell Signaling; 85061S; RRID:AB_2721019), chicken anti-TH (Millipore; AB9702; RRID:AB_570923), rabbit anti-TH (Millipore; AB152; RRID:AB_390204), goat anti-α-synuclein (R&D Systems; AF1338; RRID:AB_2192798), rabbit anti-α-synuclein (p129) (Abcam; ab51253; RRID:AB_869973), rabbit anti-vGLUT1 (Millipore; ABN1647; RRID:AB_2814811), mouse anti-CXCR4 (R&D Systems; MAB172; RRID:AB_2089399), rabbit anti-CXCL12 (Cell Signaling; 97958S), mouse anti-CD31 (Sigma; P8590; RRID:AB_2800294), goat anti-Iba1 (Wako; 019-19741; RRID:AB_839504), mouse anti-KLRB1 (Thermo Fisher; CF809736; RRID:n/a), mouse anti-CD4 (Bio-Rad; MCA1267; RRID:AB_321275), rabbit anti-IL17A (Abcam; ab79056; RRID:AB_1603584). The LSM880 confocal laser scanning microscope (Zeiss) was used to acquire images using 40x and 63x objectives. For all Z-stacks, images were acquired using optical sectioning then combined into maximum intensity projections. For quantitative histology, plot sampling was conducted on the substantia nigra by drawing a rectangular area of approximately 2mm^2^ that encompassed TH^+^ dopaminergic neurons. Three separate sections were sampled using a 40x objective. CD3^+^ T cells were then manually counted by a blinded observer in ZEN 2 Blue Edition (Zeiss). For IL-17A IR quantification, ImageJ was used to quantify positive pixels as a percentage of total image area. To verify IL-17A antibody specificity, antibody cocktails were pre-incubated with 1 µg of recombinant IL-17A (Abcam) on an orbital shaker for 1 h at room temperature prior to immunostaining.

Three-dimensional (3D) reconstruction of confocal images

Confocal image stacks were reconstructed in 3D using Imaris BitPlane v8.1.2. Each fluorescent channel was separately reconstructed using the intensity-based 3D segmentation and visualization Surface feature. Intensity thresholds for segmentation were set to match fluorescent intensities of each respective channel. Low intensity background fluorescence was filtered using the Absolute Intensity feature. After merging all channels, visualization of surfaces was performed by rotating the 3D image at a 45° angle.

Animals

Wild-type C57BL/6J and Thy1-αSyn (also known as Line 61) mice expressing human α-synuclein under the Thy1 promoter (*38, 39*) were used. The animals were housed in the American Association for Accreditation of Laboratory Animal Care-accredited animal facility of QPS Austria. Animals were housed in ventilated cages under standard conditions at a temperature of 24 °C and a 12 h light/dark cycle with ad libitum access to standard food and water. Nine male mice aged 1–6 months were analyzed per group. Animals were housed with standardized rodent bedding. The well-being of each mouse was monitored regularly. The breeding and use of Thy1-αSyn animals at QPS was approved by local ethical committees.

Animal tissue processing

Tissue was collected from 1-, 3- and 6-month-old Thy1-αSyn and wild-type mice. After deep anesthetization by intraperitoneal injection of pentobarbital, the thorax was opened and mice were transcardially perfused with 0.9% saline through the left ventricle. The brain was extracted and the hemispheres were divided at the midline. The right hemisphere was immersion-fixed in 4% paraformaldehyde in 0.1 M phosphate buffer for 2 hours at room temperature and cryoprotected overnight in 15% sucrose. Subsequently, the hemispheres were embedded in tissue freezing medium (Leica Biosystems) in cryomolds, snap-frozen in dry ice-cooled isopentane and stored at -80°C until sectioning. The hemispheres were cryosectioned in 10 µm thick sagittal slices on a Leica CM1950 cryostat, mounted on adhesive microscope slides (Leica Biosystems) and stored at -20°C until further analysis.

Immunohistochemistry and microscopy of animal tissue

Three Thy1-αSyn and three wild-type mice of three different age groups, respectively (total n = 18) were selected for immunohistochemical analysis. Of each animal, three sections of different systematically chosen brain levels were fluorescently immunolabeled according to the following protocol: cryosections were air-dried, washed for 10 minutes in PBS and blocked in 10% normal donkey serum/0.1% Triton/PBS for 1 hr at room temperature. Blocked sections were then washed 3 X 5 minutes in PBS, then incubated at 4 °C overnight in 1% normal donkey serum/PBS containing the primary antibodies: rabbit anti-mouse CD3 (Abcam; ab5690; RRID:AB_305055), rat anti-human α-synuclein (Enzo Life Sciences; ALX-804-258; RRID:AB_2270759) and sheep anti-mouse TH (Novus Biological; NB300-110, RRID:AB_10002491). The following day, sections were washed 3 X 5 min in PBS and incubated for 1 hr at room temperature in 1% normal donkey serum/PBS with the secondary antibodies: donkey anti-rabbit Alexa Fluor 555 (1:500, Abcam), donkey anti-rat Alexa Fluor 647 (1:500, Jackson Immunoresearch) and donkey anti-goat Alexa Fluor 750 (1:500, Abcam). After washing 3 X 5 minutes with PBS, nuclei were stained with DAPI (AppliChem) for 15 minutes, then sections were washed 2 X 5 minutes in PBS and an additional 5 min in aqua bidest (Fresenius) and finally covered with cover slips using Mowiol mounting medium. To assess specificity of the secondary antibodies, the experiment included an additional cryosection on which all primary antibodies were omitted. Mosaic whole slide images of the labeled sections were captured at 10x magnification using the ZEISS Axio Scan.Z1 slide scanner equipped with the ZEISS Axiocam 506 mono and a Hitachi 3CCD HV-F202SCL camera and the ZEISS ZEN 2.3 software. Uniform imaging settings were used for all samples.

Quantitative image analysis

Quantitative analyses of α-synuclein load and CD3^+^ T cells was performed with the Image-Pro 10 (Media Cybernetics). Whole hemisphere sections were defined as regions of interest (ROIs) through individual delineation, while excluding confounding factors such as air bubbles, folds or large artifacts. Thresholds and morphological filters were set to automatically detect immunoreactive objects in both the human α-synuclein and the CD3 channel of 3-5 images. To avoid the inclusion of erroneously recognized CD3^+^ objects (e.g. small artifacts that matched the threshold parameters) in the further evaluation, all automatically detected objects were manually rescreened and rejected, if necessary. Human α-synuclein and CD3^+^ object densities were calculated by normalizing the number of objects to the corresponding ROI size. Statistical analysis was performed with Prism v8.4.3 (GraphPad).

Flow cytometry

Flow cytometry was conducted using an LSRFortessa (BD Biosciences). A panel consisting of antibodies conjugated to four different fluorophores was used to quantify or sort activated T cells. Antibodies used were: CD3 BV650 (BD Biosciences), CD38 PE (Biolegend), CD69 FITC (Biolegend) and HLA-DR APC-Cy7 (Biolegend). For sorting, SYTOX Blue dead cell dye (Thermo Fisher) was used to sort live cells. For each experiment, a compensation matrix was developed using singly stained and unstained controls or fluorescent beads, and all analysis was conducted in Cytobank.

Cell stimulation

PBMCs were thawed and plated at a density of 1x10^6^ cells per well in a 24 well plate. Media consisted of RPMI with 5% human AB serum (Sigma Aldrich) and 1X penicillin-streptomycin. After overnight incubation at 37°C with 20 U/mL interleukin-2, cells were stimulated either with a pool of α-synuclein peptides or single peptides. Peptides were synthesized by JPT Peptides and had >90% purity by high performance liquid chromatography. Lyophilized peptides were resuspended at 1 µg/µl in phosphate buffered saline. Peptides were added to PBMCs at 1:100 dilution in 250 μl of media (final concentration 2.5 µg/mL). Unstimulated cells received an equal volume of phosphate buffered saline. Cells were then incubated an additional 6 days before performing intracellular flow cytometry analysis. 100 µl of media and Interleukin-2 (20 U/mL) were added every two days to maintain cell viability.

Drop-seq of CSF cells

For scRNAseq of CSF cells, FASTQ files from a NovaSeq 6000 (Illumina) were generated by Novogene. For CSF scTCRseq, base call files from a NextSeq550 Sequencer (Illumina) were generated in house. Single cell V(D)J and 5’ gene expression analysis (10X Genomics) was used for scTCRseq. Cellranger version 3.0.2 was used to generate gene expression matrices. The cellranger mkfastq pipeline generated FASTQ files for both the 5’ expression libraries and the V(D)J libraries. Reads from the 10x v2 5’ paired library were mapped to human genome GRCh38 3.0.0. Reads from the 10x V(D)J kit were mapped to the vdj-GRCh38 alts ensemble 2.0.0 available from 10X Genomics. The 5’ gene expression libraries were then analyzed with cellranger count pipeline and the resulting expression matrix was used for further analysis in the ‘Seurat’ package v3.0. The V(D)J FASTQ files were analyzed with the cellranger vdj pipeline, which produced single cell V(D)J sequences and determined clonotypes. Clonotypes were determined by grouping of cell barcodes that shared the same set of productive CDR3 nucleotide sequences. The sequences of all contigs from all cells within a clonotype were then assembled to produce a clonotype consensus sequence. Clonality was integrated into the Seurat gene expression analysis by adding clonality information to the metadata.

Clustering of CSF cells

Individual sample expression matrices were loaded into R using the function Read10x under the ‘Seurat’ package v1.2-15. The expression matrix for each sample was merged into one Seurat object using the CreateSeuratObject and MergeSeurat functions. Seurat package v3.0 was utilized for filtering, variable gene selection, normalization, scaling, dimensionality reduction, clustering and visualization. For CSF cells, genes were excluded if they were expressed in less than 10 cells and cells were excluded if they expressed fewer than 200 genes. Cells expressing more than 2500 genes, more than 10,000 unique molecular identifiers (UMIs), and more than 10% mitochondrial genes were excluded. Regularized negative binomial regression using the sctransform normalization method was used to normalize, scale, select variable genes and regress out sequencing and experimental batch, mitochondrial mapping percentage, and the number of UMIs. Following PCA, principle components were selected for clustering the cells

PBMC preparation

Vials of frozen PBMCs were rapidly thawed in a 37°C water bath for ~2 minutes. Vials were removed when a small ice crystal was left. Thawed PBMCs were quenched with 13 ml 37°C pre-warmed 1X PBS supplemented with 10% fetal bovine serum (FBS). Cells were centrifuged at 300 x g for 10 minutes at room temperature. The supernatant was removed, and cell pellet was resuspended in 3 ml 1X PBS containing 10% FBS, passed through a 40μm cell strainer, then centrifuged at 300 x g for 10 minutes at room temperature. Dead cells were removed by magnetic beads purification (Miltenyi Biotech) according to the manufacturer’s protocol. Cells were resuspended with cell resuspension buffer at a concentration of 1,000 viable cells/μl.

PBMC scRNAseq

The DNBelab C Series Single-Cell Library Prep Set (MGI) was utilized. In brief, single-cell suspensions were used for droplet generation, emulsion breakage, bead collection, reverse transcription, and cDNA amplification to generate barcoded libraries. Indexed scRNAseq libraries were constructed according to the manufacturer’s protocol. DNA nanoball-based libraries were sequenced by the ultra-high-throughput DIPSEQ T1 sequencer at China National GeneBank. The read structure was paired-end with Read 1, covering 30 bases inclusive of 10-bp cell barcode 1, 10-bp cell barcode 2 and 10-bp unique molecular identifier (UMI), and Read 2 containing 100 bases of transcript sequence, 10-bp sample index.

PBMC scRNAseq data processing

Raw sequencing reads from DIPSEQ T1 sequencer were filtered and demultiplexed using PISA (version 0.2). Reads were aligned to hg38 genome using STAR (version 2.7.4a) and sorted by sambamba (version 0.7.0). Genes were annotated according to GENCODE 32. Cell versus gene UMI count matrix was generated with PISA.

Analysis of scRNAseq data

For differential expression analysis, markers for each cluster were determined by comparing the cells of each cluster to all other cells using the FindMarkers function in Seurat with the Model-based Analysis of Single Cell Transcriptomics (MAST) algorithm from the R package ‘MAST’ version 1.8.2. For all comparisons between groups and clusters, only genes expressed by at least 10 or 50% of cells were included. The R package ‘ggplot2’ version 3.1.0 was used to plot the results of the differential expression analysis, showing the average log fold change of each gene and the -log10 of the Benjamini-Hochberg (BH)-adjusted *P* value. Mitochondrial and ribosomal genes were filtered for volcano plots. For pathway analysis, the R package ‘topGO’ was used to perform pathway analysis on the top 500 genes identified from differential expression analysis (BH < 0.001) and with all genes in the dataset as background. Enriched ontology terms for biological processes were identified using Fisher’s exact test. Z-scores for each pathway were calculated using the R package ‘GOPlot’.

Statistical Methods

All statistical analyses were performed using Prism. All values are expressed as the mean ± SEM. Differences in means between two groups were analyzed using unpaired two-sided heteroscedastic t-tests with Welch’s correction. For regression analyses, significance of the difference between datasets was measured by analysis of covariance. Differences in means among multiple data sets were analyzed using one- or two-way analysis of variance (ANOVA). When ANOVA showed significant differences, pair-wise comparisons between means were tested by Tukey’s multiple comparisons test. For scRNAseq analyses, we corrected for multiple comparisons and report adjusted *P* values using Benjamini-Hochberg correction. For pathway analyses, Fisher’s exact test was used with Bonferroni correction for multiple testing.


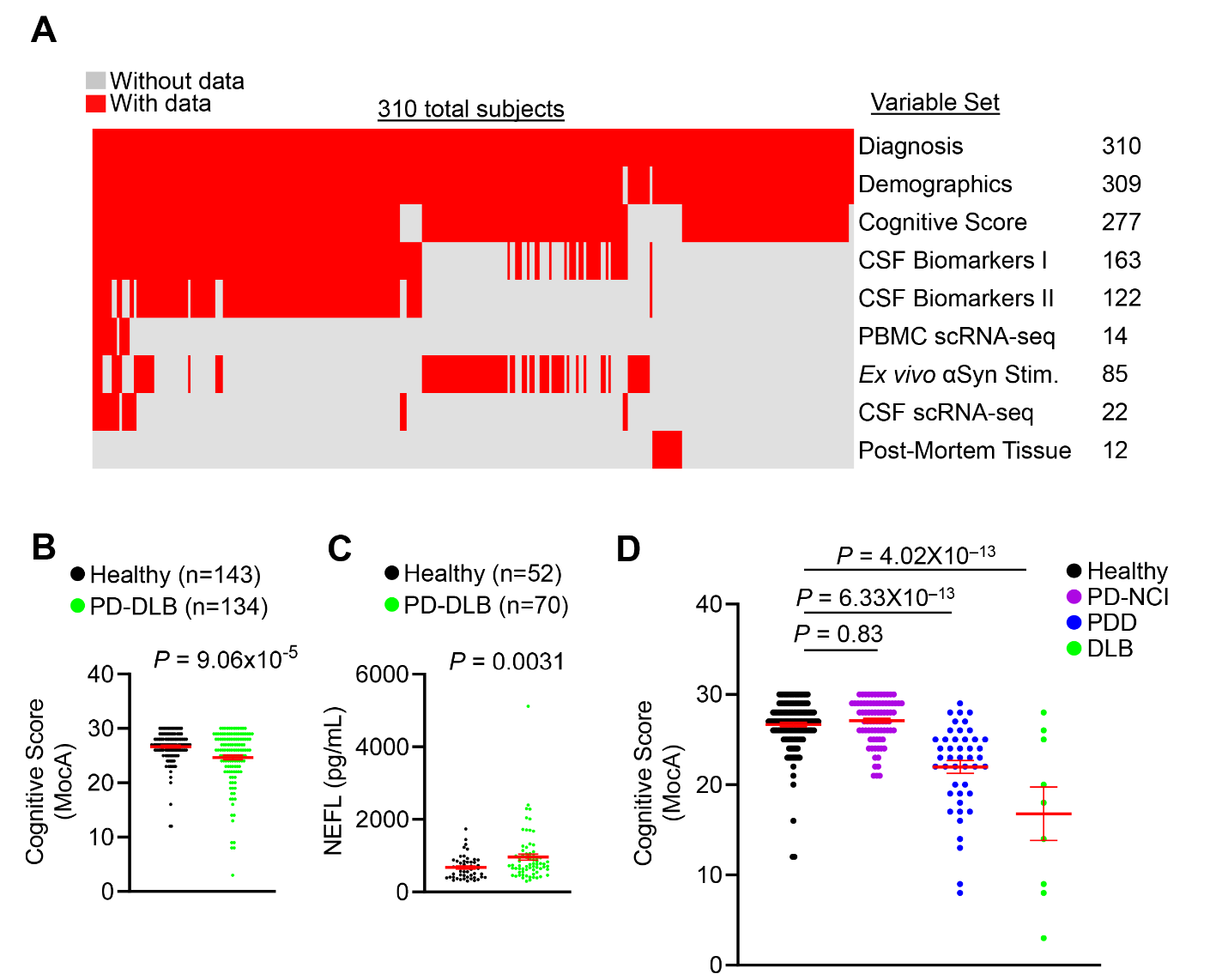


Fig. S1.

(A) Schematic depicting data collected for all 310 study subjects. (B) Cognitive measures of living study subjects shows significantly lower MoCA scores among PD-DLB subjects. (C) Levels of CSF NEFL protein showing significantly higher levels among PD-DLB subjects. (D) Parsing of study subjects into PD-NCI, PDD and DLB shows lower MoCA scores among PDD and DLB subjects.


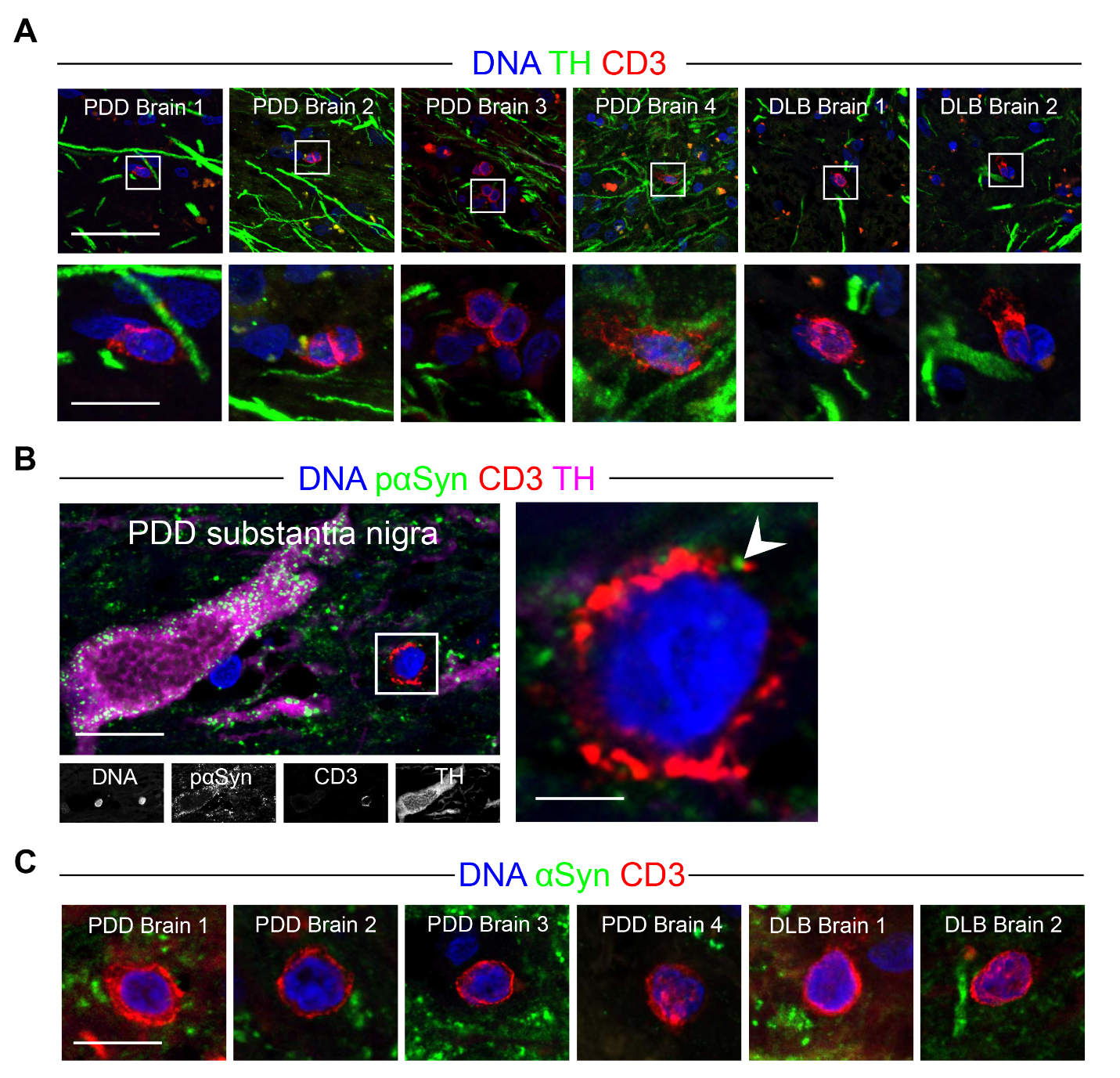


Fig. S2.

(A) Representative confocal images of CD3^+^ T cells in post-mortem PDD and DLB brains. Scale bar = 100 μm. Insets show CD3^+^ T cells adjacent to TH^+^ dopaminergic neuronal processes. Inset scale bar = 10 μm. (B) A TH^+^ dopaminergic neuron laden with α-synuclein with an adjacent CD3^+^ T cell in PDD substantia nigra. Scale bar = 10 μm. Inset shows α-synuclein in close proximity to CD3 (arrowhead). Inset scale bar = 2 μm. Similar results were observed in 6/7 LBD brains. (C) Representative CD3^+^ T cells adjacent to α-synuclein deposits in PDD and DLB brains. Scale bar = 10 μm.


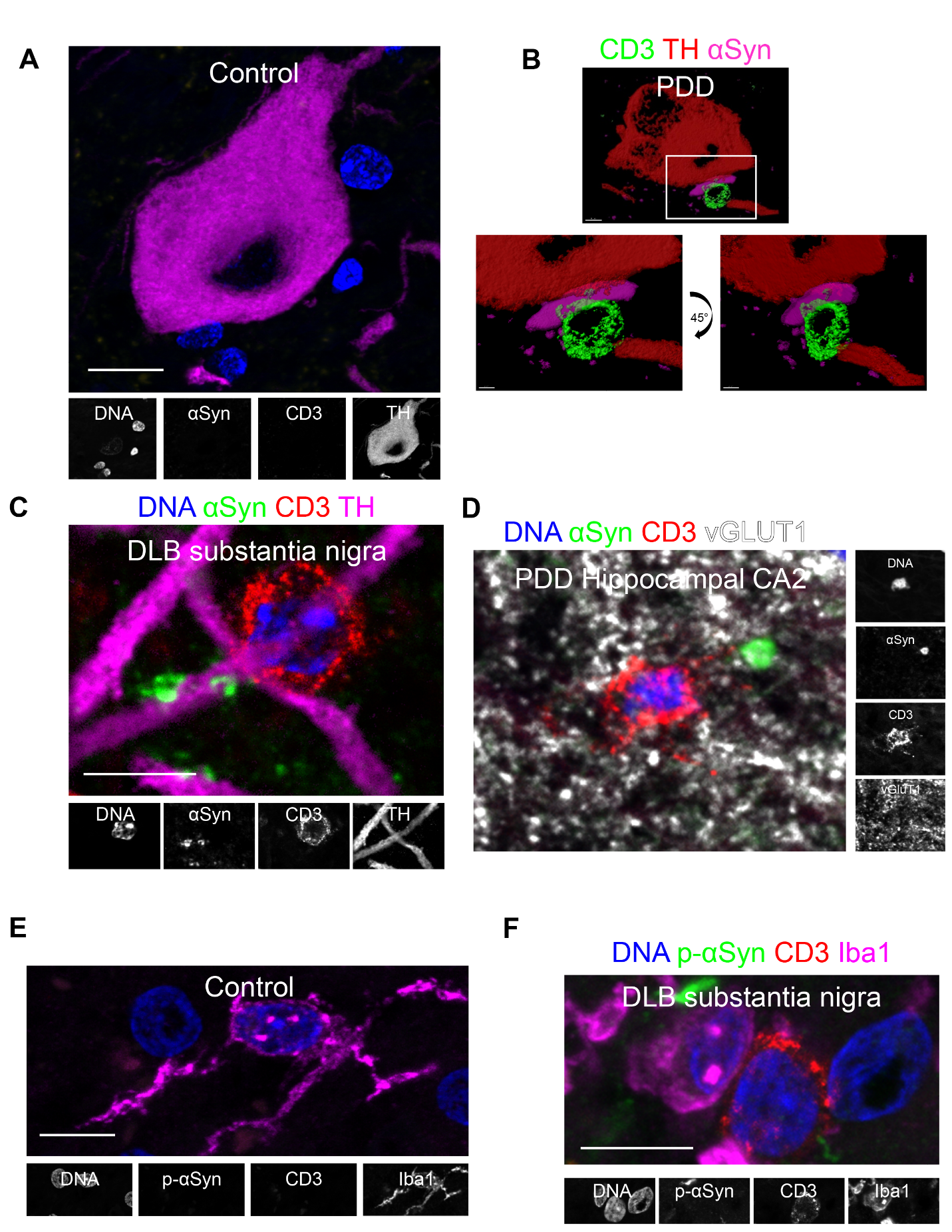


Fig. S3.

(A) Confocal image of a TH^+^ dopaminergic neuron in control (non-neurologic disease) substantia nigra (B) 3D reconstruction of the confocal image shown in Fig. 1D showing a CD3^+^ T cell extending filopodia-like extensions into an α-synuclein^+^ Lewy neurite in PDD substantia nigra. (C) Representative confocal image of a CD3^+^ T cell adjacent to a Lewy neurite in DLB substantia nigra. (D) Lewy body adjacent to vGLUT1^+^ glutamatergic neurons in the hippocampal CA2 region in PDD. Scale bar = 10 μm. Similar results were observed in 6/7 LBD brains. (E) Confocal image of an Iba1^+^ innate immune cell control (non-neurologic disease) substantia nigra (F) Representative confocal image of a CD3^+^ T cell bound to an Iba1^+^ innate immune cell adjacent to phosphorylated α-synuclein.

Fig. S4.

CD3^+^ T cells adjacent to α-synuclein deposits in the midbrain of Thy1-αSyn mice.


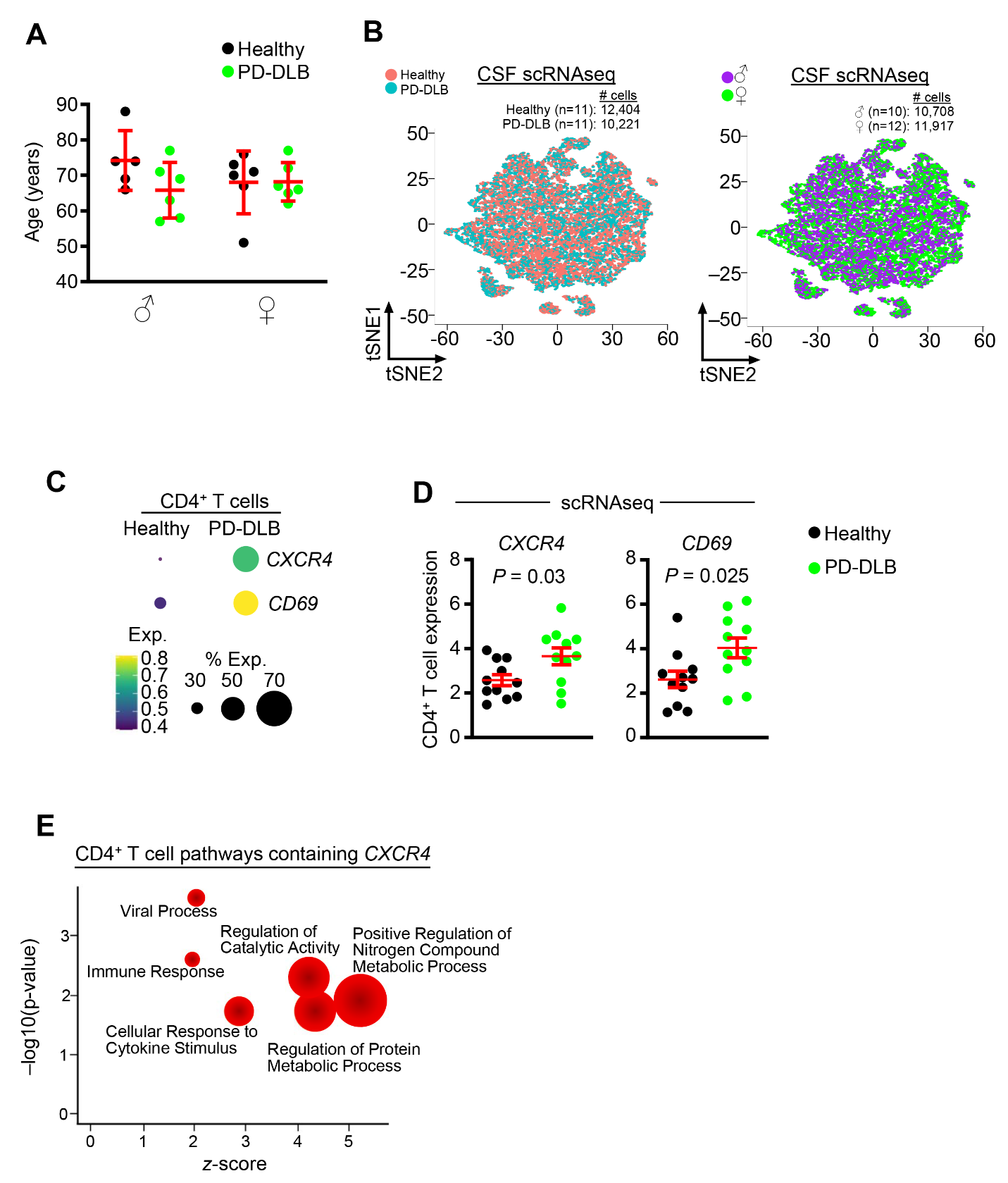


**Fig. S5.**

(A) Healthy and PD-DLB subjects were age- and sex-matched in scRNAseq experiments. (B) tSNE plots showing no impact of group or sex on scRNAseq cell clusters. (C) Dot plot showing increased expression of *CXCR4* and *CD69* in PD-DLB versus healthy CSF CD4^+^ T cells. (D) Analysis of individual subjects’ CSF CD4^+^ T cell CXCR4 and CD69 expression showing significantly higher levels in the PD-DLB group. (E) Pathway analysis of differentially expressed genes between healthy and PD-DLB CSF CD4^+^ T cells shows changes to metabolism and response to cytokine stimulus. Pathways containing *CXCR4* are shown.


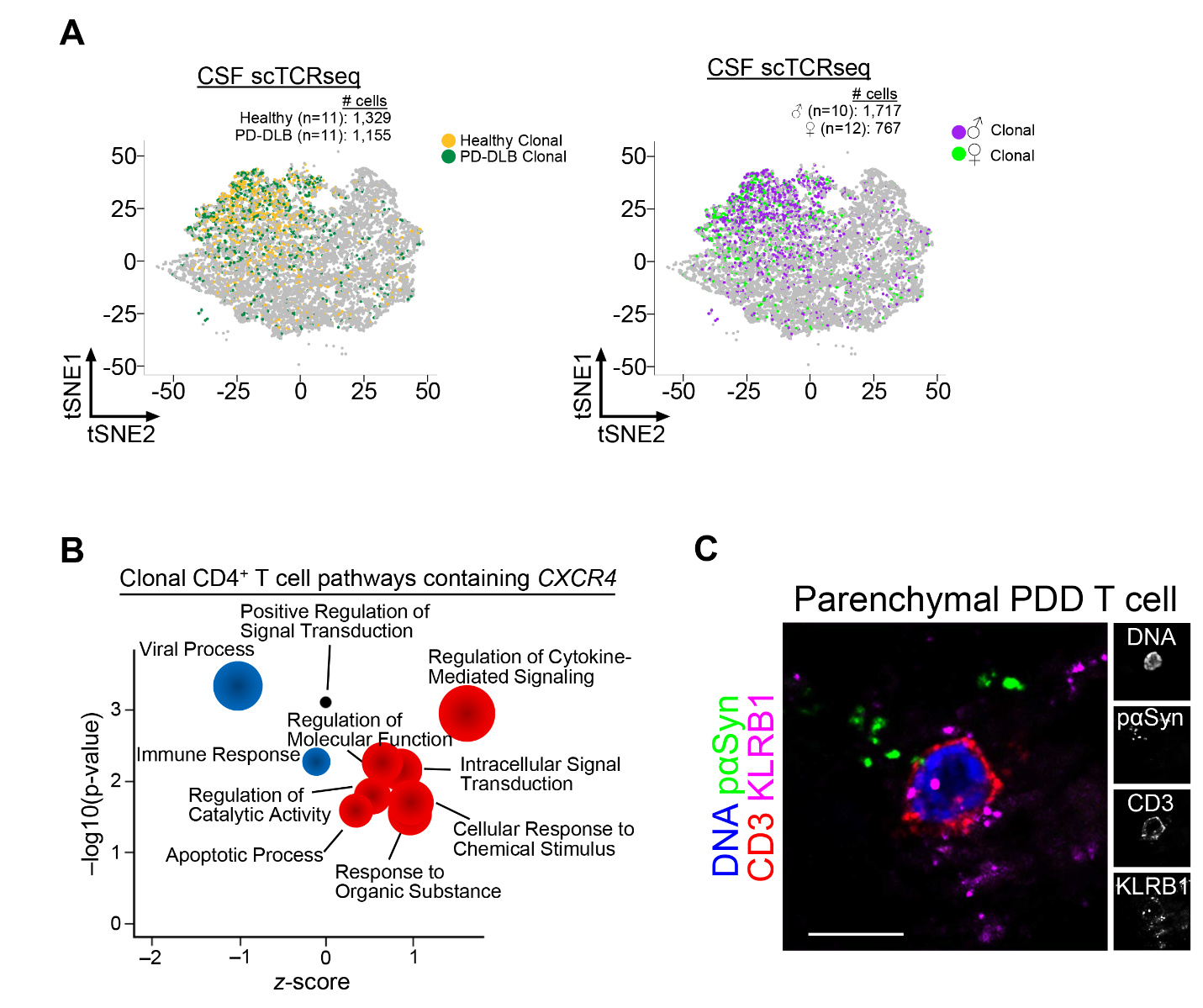


Fig. S6.

(A) tSNE plot showing no impact of group or sex on the distribution of clonal CD4^+^ T cells. (B) Pathway analysis of differentially expressed genes between healthy and PD-DLB clonal CSF CD4^+^ T cells shows changes to cytokine-mediated signaling and signal transduction. (C) Confocal image of a CD3^+^KLRB1^+^ T cell in close proximity to α-synuclein deposits in the parenchyma of PDD substantia nigra. Similar results were observed in 6/7 LBD brains.


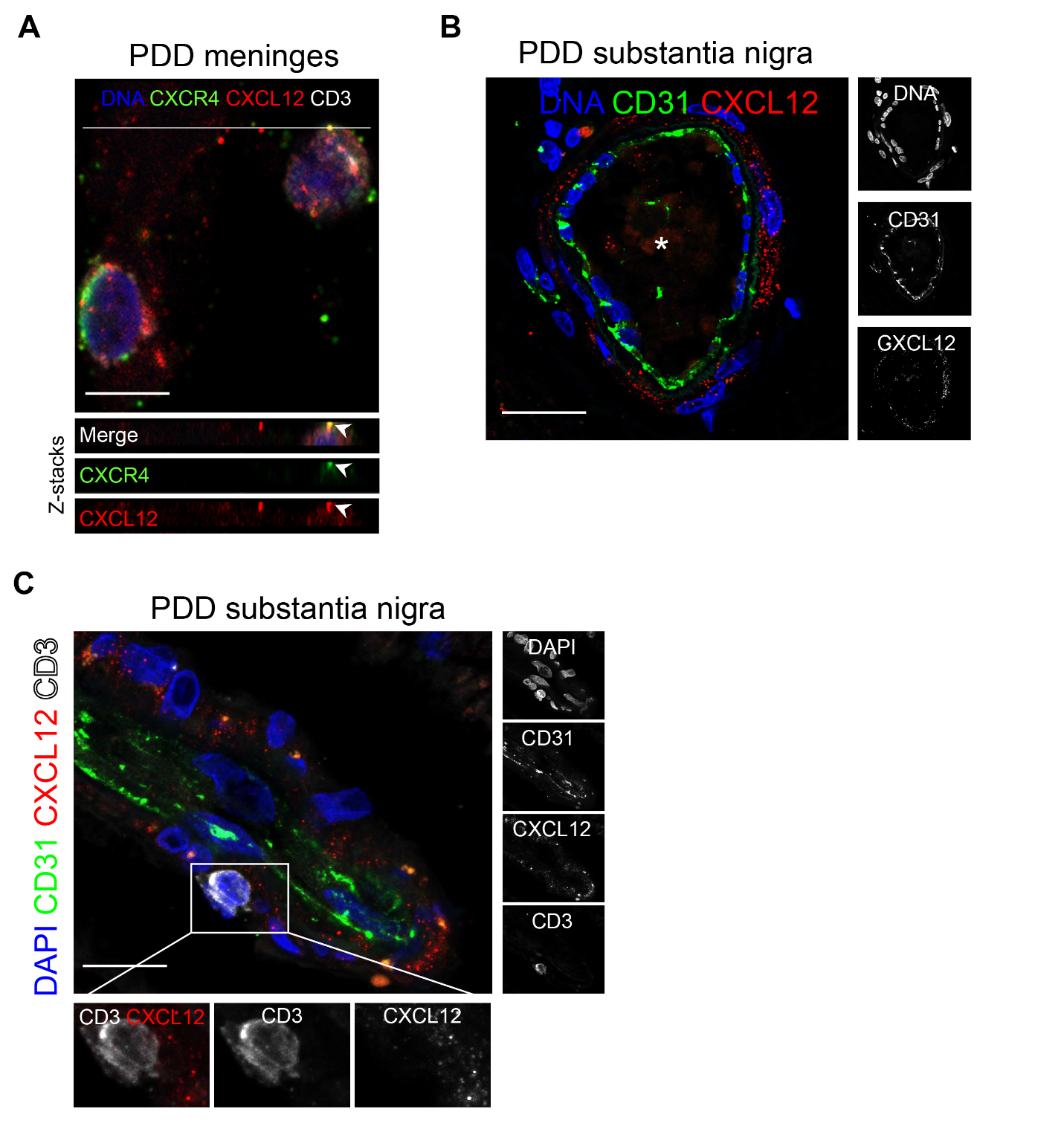


Fig. S7.

(A) Confocal image of CD3^+^CXCR4^+^CXCL12^+^ cells in the PDD meninges. Z-stacks show CXCL12 colocalized with CXCR4 on the cell surface. The z-stack plane is indicated by a thin white line. Scale bar = 10 μm. (B) Localization of CXCL12 cytokine to CD31^+^ brain endothelial cells. (C) A CD3^+^ T cell colocalized with CXCL12 cytokine in the perivascular space adjacent to a CD31^+^ blood vessel in the PDD substantia nigra. Similar results were observed in 6/7 LBD brains.


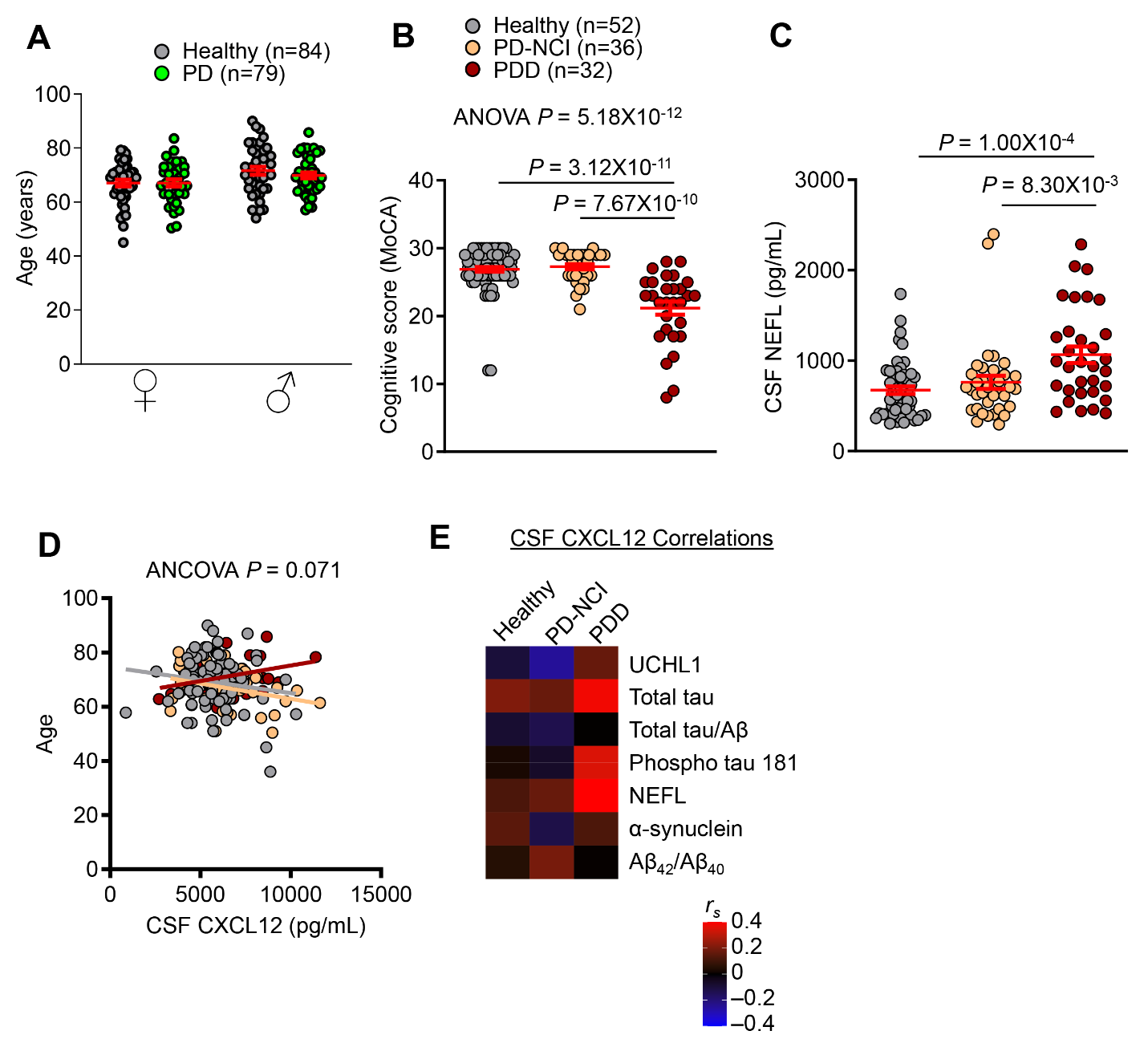


Fig. S8.

(A) Healthy and PD subjects assessed by CSF biomarkers were age- and sex-matched. (B) Cognitive scores of healthy vs. PD subjects show lower MoCA scores in PDD vs. healthy and PD-NCI subjects. (C) Higher levels of CSF NEFL in PDD patients vs. healthy and PD-NCI subjects. (D) Lack of correlation between age and CSF CXCL12 levels in all three groups. (E) Spearman correlations (r_s_) between CXCL12 and disease biomarkers are shown. Note the high correlation between NEFL and CXCL12 in PDD patients.


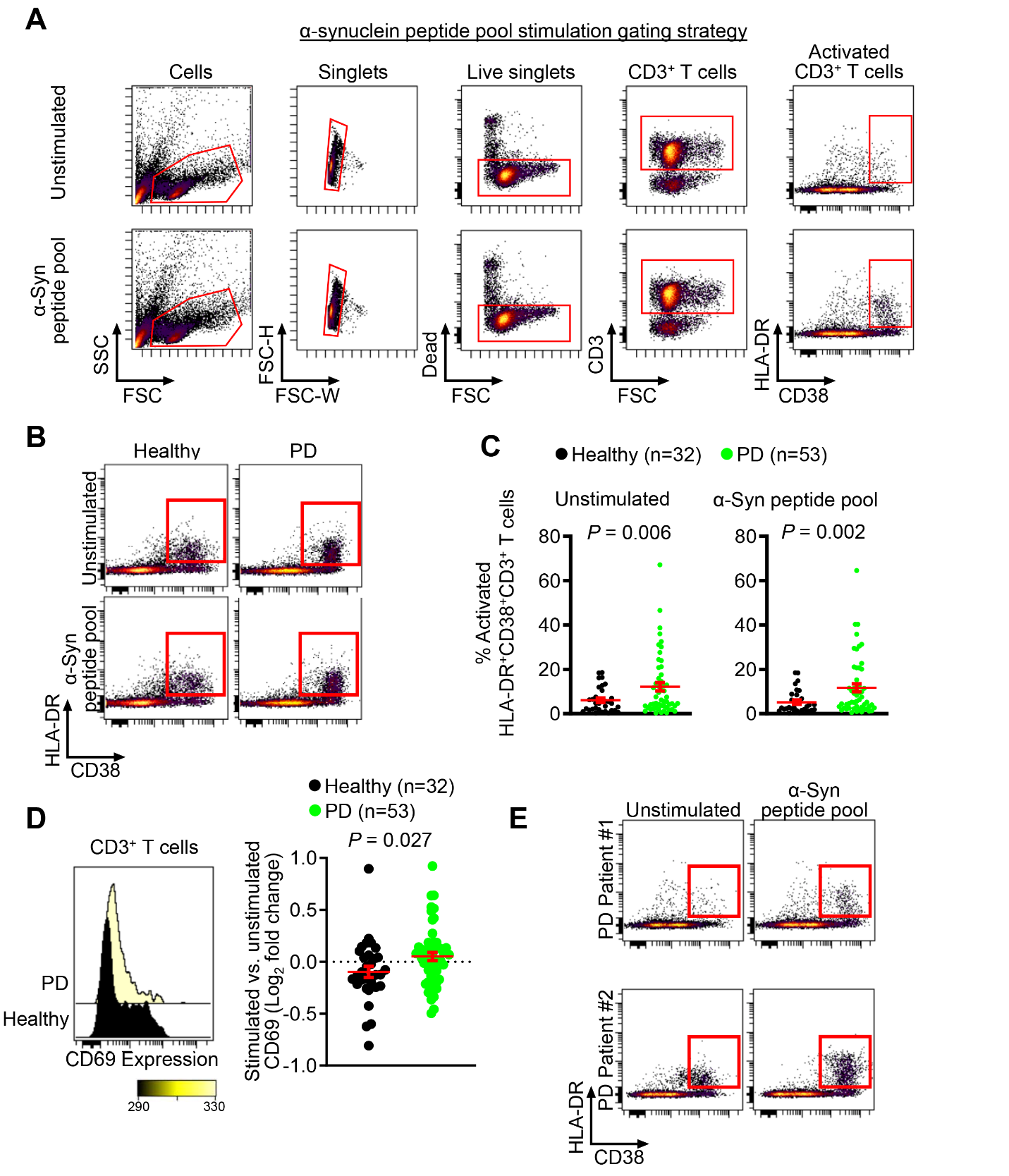


Fig. S9.

(A) Flow cytometry gating strategy for quantifying and sorting activated T cells following α-synuclein peptide pool incubation. (B) Representative flow cytometry plots and (C) quantification showing increased T cell activation in unstimulated and stimulated PD T cells. (D) Histogram and quantification of CD69 expression of T cells following α-synuclein peptide pool incubation showing increased expression in PD. (E) Two PD patients showed pronounced T cell activation by flow cytometry following α-synuclein peptide pool incubation.


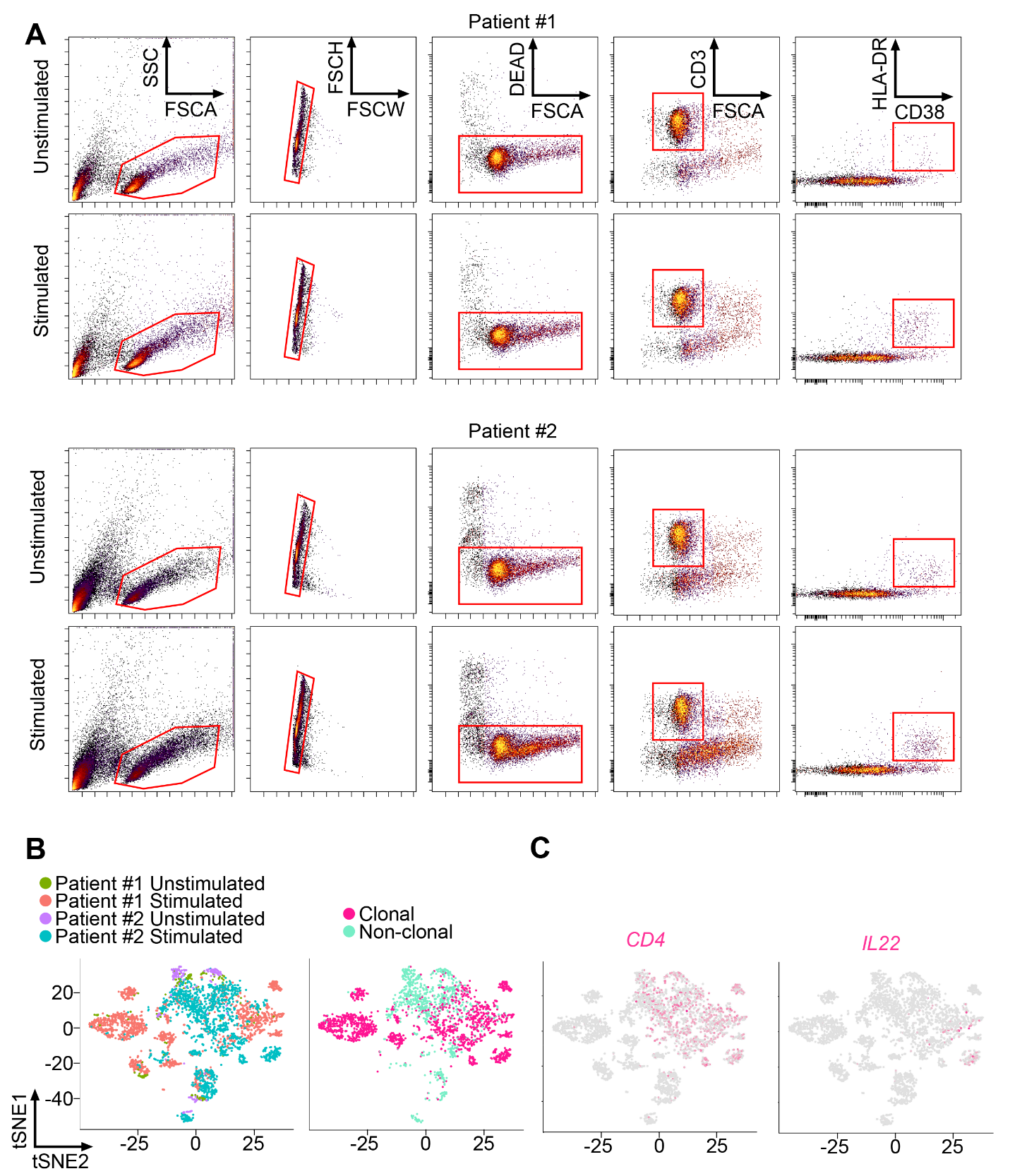


Fig. S10.

(A) Flow cytometry gating strategy for sorting activated T cells following incubation of Patient #1 and Patient #2 PBMCs with an antigenic α-synuclein peptide. (B) tSNE plots showing unstimulated and stimulated populations of activated T cells (at left) and the distribution of clonal T cells (at right) (C) tSNE plots showing expression of *CD4* and *IL22* in cells incubated with an antigenic α-synuclein peptide. Note the colocalization of expression with IL17A expressing clonotypes from Fig. 4E.


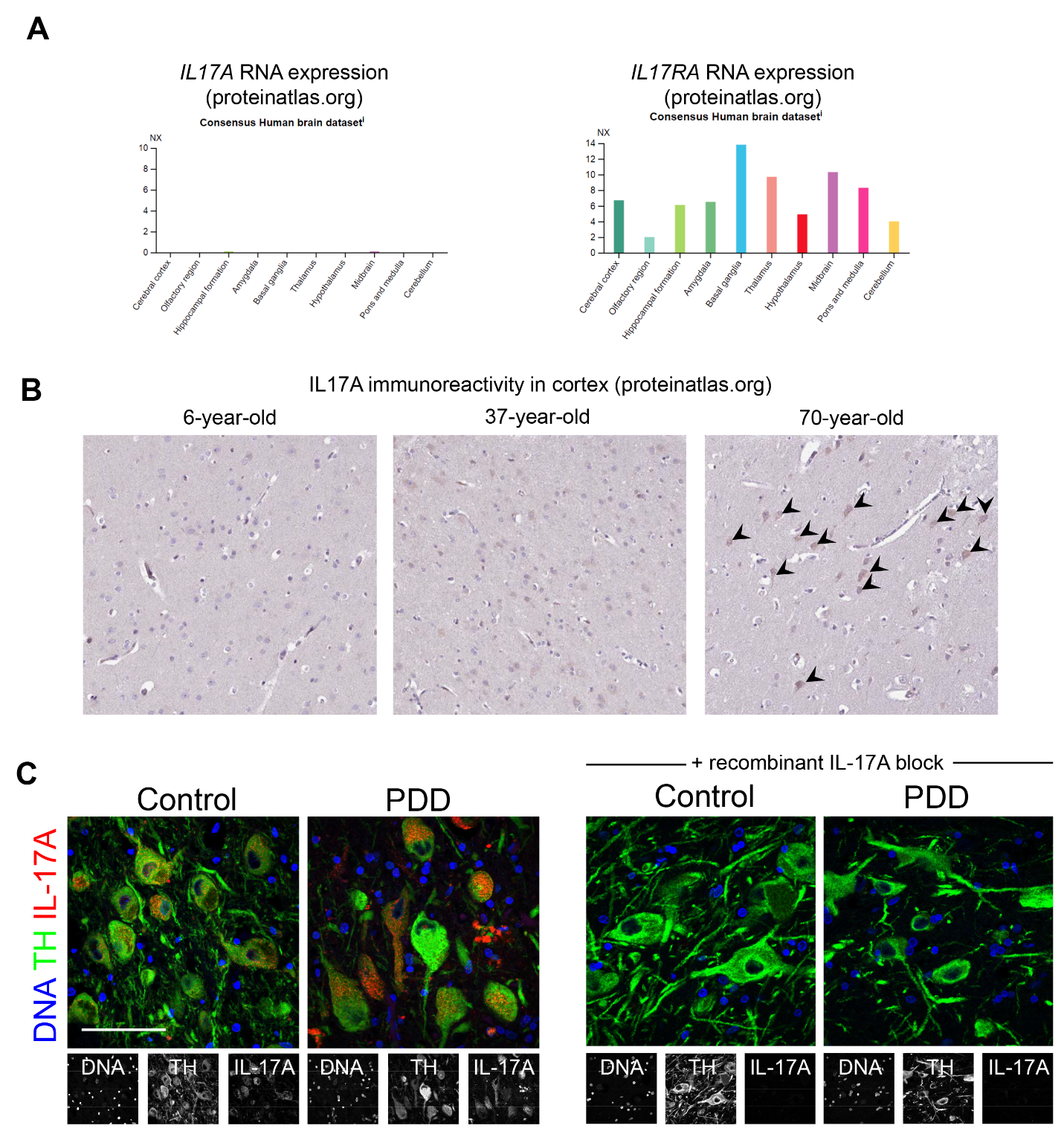


Fig. S11.

(A) Plots of *IL17A* and *IL17RA* RNA expression in various human brain regions from proteinatlas.org. Human brains appear to lack *IL17A* expression but express *IL17RA* at high levels in the midbrain, suggesting an external source of IL-17A in the human brain. (B) Histology images from proteinatlas.org showing an age-dependent increase in IL17A protein in neurons. (C) Representative confocal images of IL-17A signal in the substantia nigra (at left). This signal is ablated by pre-incubation of IL-17A antibody with recombinant IL-17A (at right). Similar results were observed in 6/7 LBD brains.


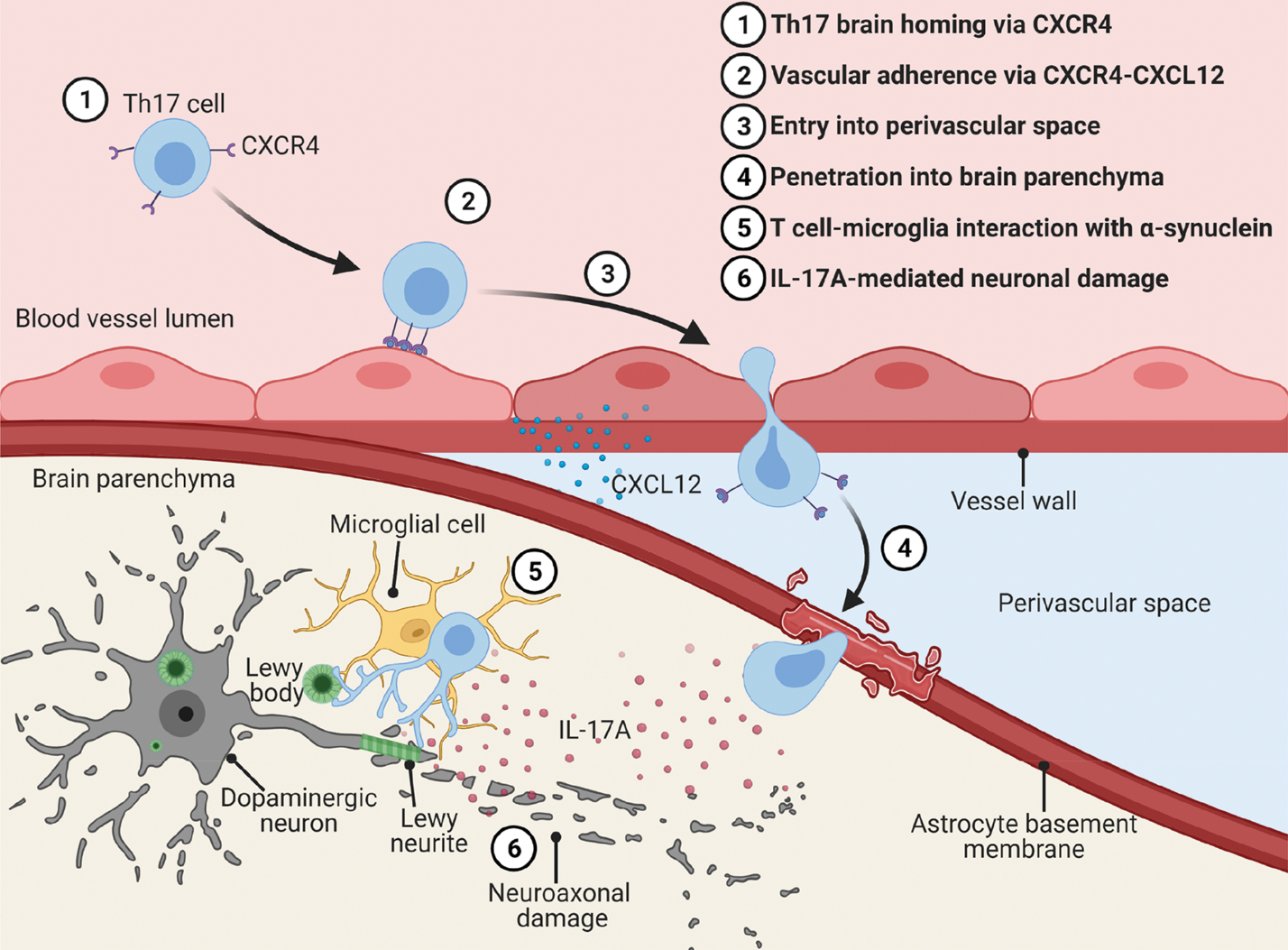


**Fig. S12.**

Schematic summarizing the potential mechanism of Th17 cell brain homing and neuronal damage in LBD. CXCR4 mediates homing to the brain via cerebrovasculature expression of CXCL12. Upon traversing the astrocytic basement membrane, Th17 cells encounter microglial cells and α-synuclein deposits such as Lewy bodies and Lewy neurites. Th17 cells secrete pro-inflammatory IL-17A to enter the brain and induce neuroaxonal injury, resulting in dopaminergic neuron loss in LBD.


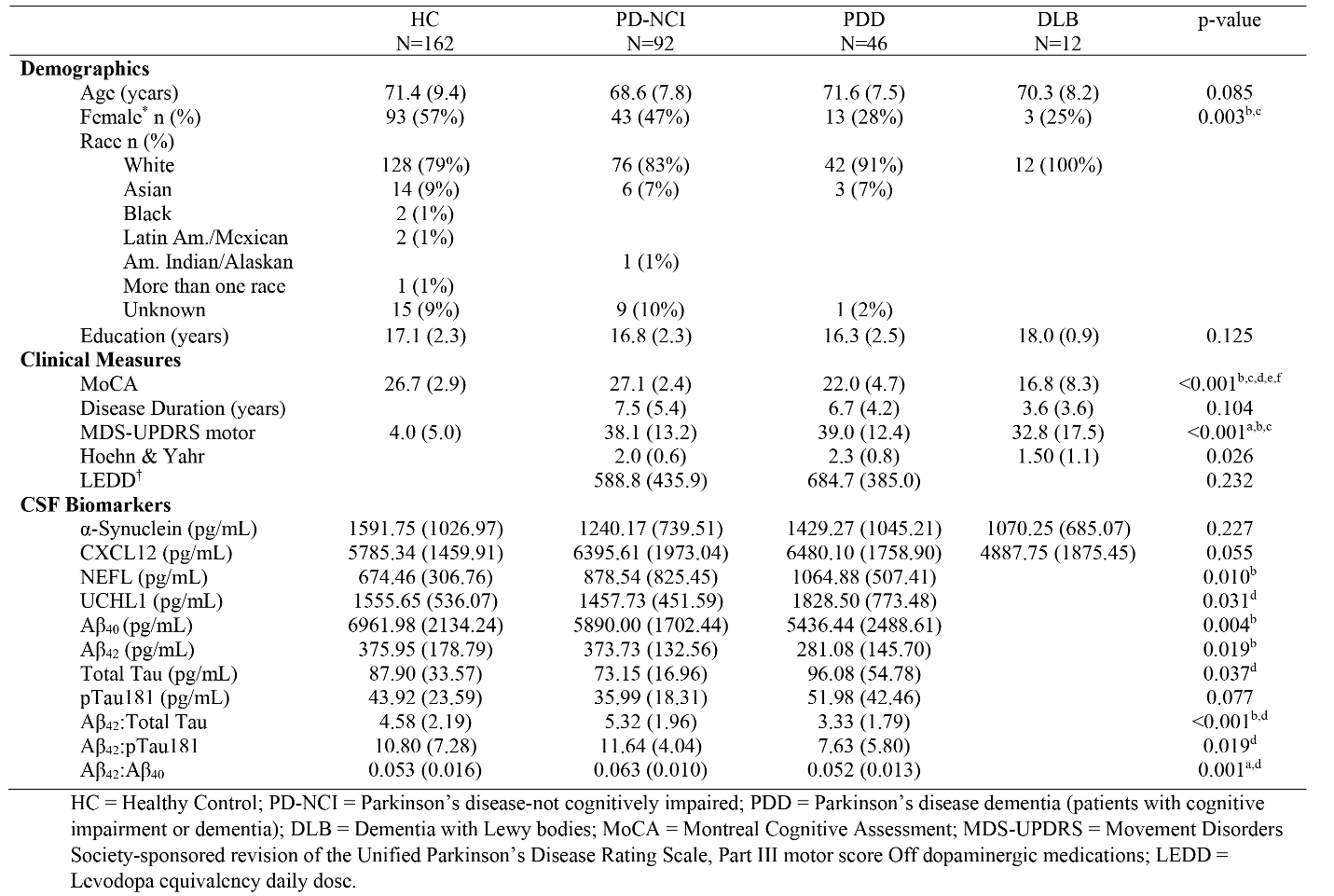


**Table S1. Participant Demographics.** Table depicts mean (standard deviation) for the demographic and clinical data with *p* values computed using one-way ANOVA or *χ^2^ test or †Student’s t-test as appropriate.

Bonferroni post-hoc corrections p≤0.05 between:

^a^ HC vs. PD

^b^ HC vs. PD-CI

^c^ HC vs. DLB

^d^ PD vs. PD-CI

^e^ PD vs DLB

^f^ PD-CI vs. DLB

Data S1. Study Subjects and Biomarker Data (separate file)

Demographic and biomarker data for all study subjects from Fig. S1 and Table S1.

Data S2. CSF Immune Cell Differential Expression (separate file)

Differential expression data of CSF immune cells between healthy and PD-DLB CSF from Fig. 2C.

Data S3. CSF Clonal CD4^+^ T Cell Differential Expression (separate file)

Differential expression data of CSF clonal CD4^+^ T cells between healthy and PD-DLB CSF from Fig. 3B.

Data S4. Alpha Synuclein Stimulation Differential Expression (separate file)

Differential expression data between stimulated and unstimulated activated (HLA-DR^+^CD38^+^) CD3^+^ T cells of PD patients from Fig. 4D.
